# Canagliflozin primes antitumor immunity by triggering PD-L1 degradation in endocytic recycling

**DOI:** 10.1172/JCI154754

**Published:** 2023-01-03

**Authors:** Ling Ding, Xi Chen, Wenxin Zhang, Xiaoyang Dai, Hongjie Guo, Xiaohui Pan, Yanjun Xu, Jianguo Feng, Meng Yuan, Xiaomeng Gao, Jian Wang, Xiaqing Xu, Sicheng Li, Honghai Wu, Ji Cao, Qiaojun He, Bo Yang

**Affiliations:** 1 Zhejiang Province Key Laboratory of Anti-Cancer Drug Research, Institute of Pharmacology and Toxicology, College of Pharmaceutical Sciences, and; 2 Center of Drug Safety Evaluation and Research, Zhejiang University, Hangzhou, China.; 3 Department of Medical Thoracic Oncology and; 4 Institute of Basic Medicine and Cancer, The Cancer Hospital of the University of Chinese Academy of Sciences, Zhejiang Cancer Hospital, Chinese Academy of Sciences, Hangzhou, China.; 5 The Innovation Institute for Artificial Intelligence in Medicine and; 6 Cancer Center of Zhejiang University, Hangzhou, China.

**Keywords:** Immunology, Cancer immunotherapy

## Abstract

Understanding the regulatory mechanisms of PD-L1 expression in tumors provides key clues for improving immune checkpoint blockade efficacy or developing novel oncoimmunotherapy. Here, we showed that the FDA-approved sodium-glucose cotransporter-2 (SGLT2) inhibitor canagliflozin dramatically suppressed PD-L1 expression and enhanced T cell–mediated cytotoxicity. Mechanistic study revealed that SGLT2 colocalized with PD-L1 at the plasma membrane and recycling endosomes and thereby prevented PD-L1 from proteasome-mediated degradation. Canagliflozin disturbed the physical interaction between SGLT2 and PD-L1 and subsequently allowed the recognition of PD-L1 by Cullin3^SPOP^ E3 ligase, which triggered the ubiquitination and proteasome-mediated degradation of PD-L1. In mouse models and humanized immune-transformation models, either canagliflozin treatment or SGLT2 silencing significantly reduced PD-L1 expression and limited tumor progression — to a level equal to the PD-1 mAb — which was correlated with an increase in the activity of antitumor cytotoxic T cells. Notably, prolonged progression-free survival and overall survival curves were observed in the group of PD-1 mAb–treated patients with non–small cell lung cancer with high expression of SGLT2. Therefore, our study identifies a regulator of cell surface PD-L1, provides a ready-to-use small-molecule drug for PD-L1 degradation, and highlights a potential therapeutic target to overcome immune evasion by tumor cells.

## Graphical abstract

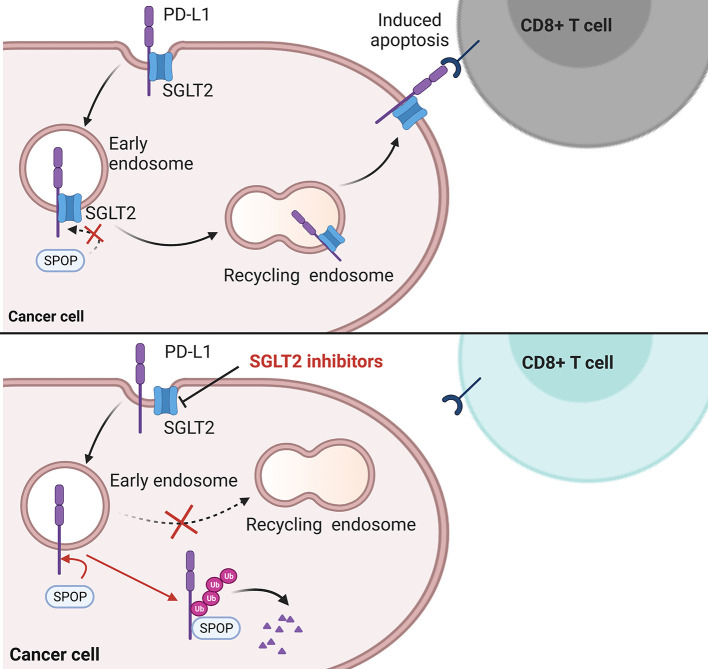

## Introduction

The remarkable clinical responses of PD-L1/PD-1 blockade confirm the crucial role of the PD-L1/PD-1 axis in tumor immune escape and led to the approval of PD-L1/PD-1 inhibitors by the FDA in more than 10 cancer indications (1). Now, understanding the mechanisms regulating PD-L1 expression has attracted interest for several reasons. First, although clinical benefits have been seen in several different malignancies — including, but not limited to, melanoma, lung, kidney, and bladder cancers — determining which patients derive benefit from PD-1/PD-L1 directed immunotherapy remains an important clinical question. Patients whose tumors overexpress PD-L1 by IHC have improved clinical outcomes with anti–PD-L1/PD-1 therapy, but the presence of robust responses in some patients with low levels of expression of these markers complicates the issue of PD-L1 as an exclusionary predictive biomarker (2, 3). An improved understanding of PD-L1 expression will better elucidate which patients derive benefit from these inhibitors. Second, for patients with poor response, increasing PD-L1 expression in the tumor could be an efficient strategy to sensitize the monoclonal antibody (1). Studies have validated histone deacetylase (HDAC) inhibitors and poly (ADP-ribose) polymerase (PARP) inhibitors can upregulate the expression of PD-L1 and enhance the efficacy of PD-1 mAbs (4–6). Clinical trials have also been designed to evaluate the effect of PD-1/PD-L1 blockade combined with epigenetic agents and PARP inhibitors among different cancer types (7, 8). Third, small molecules inhibitors targeting PD-1/PD-L1 are highly desirable, given the limitations of the existing antibody-based therapies, including poor response rates, immune-related adverse events, and the intravenous route of dosing (9, 10). Methods have also been developed to suppress PD-L1 expression by targeting its gene transcription, posttranscriptional and posttranslational modifications. For example, JQ1 prevents BRD4 from binding to the *CD274* promoter region and inhibits its transcription (11). eFT508 reduces the phosphorylation level of eIF4E to suppress PD-L1 protein translation (12). Curcumin inhibits CSN5 activity to promote the ubiquitination-mediated degradation of PD-L1(13). These compounds have also been shown to inhibit tumor growth efficiently in animal models. However, they are not suitable for further development in the clinic, due to their toxicity or off-target effect.

Here we report that the antidiabetes drug canagliflozin suppresses tumor growth by reducing the expression of PD-L1 in an on-target manner. Mechanistically, sodium-glucose cotransporter-2 (SGLT2) maintains PD-L1 stability in endocytic recycling through direct interaction. Canagliflozin disrupts the interaction between SGLT2 and PD-L1, and further promotes the interaction between PD-L1 and the E3 ubiquitin ligase SPOP. In mouse models and 2 humanized immune-transformation models, canagliflozin treatment significantly reduced PD-L1 expression, increased the activity of antitumor cytotoxic T cells, and eventually mediated tumor regression. Furthermore, the expression of SGLT2 positively correlates with the level of PD-L1 in non–small cell lung cancer (NSCLC) tissues, and high SGLT2 level predicts poor prognosis in NSCLC. Therefore, canagliflozin has the potential to act as an immunooncology drug by targeting PD-L1.

## Results

**Figure 1:**
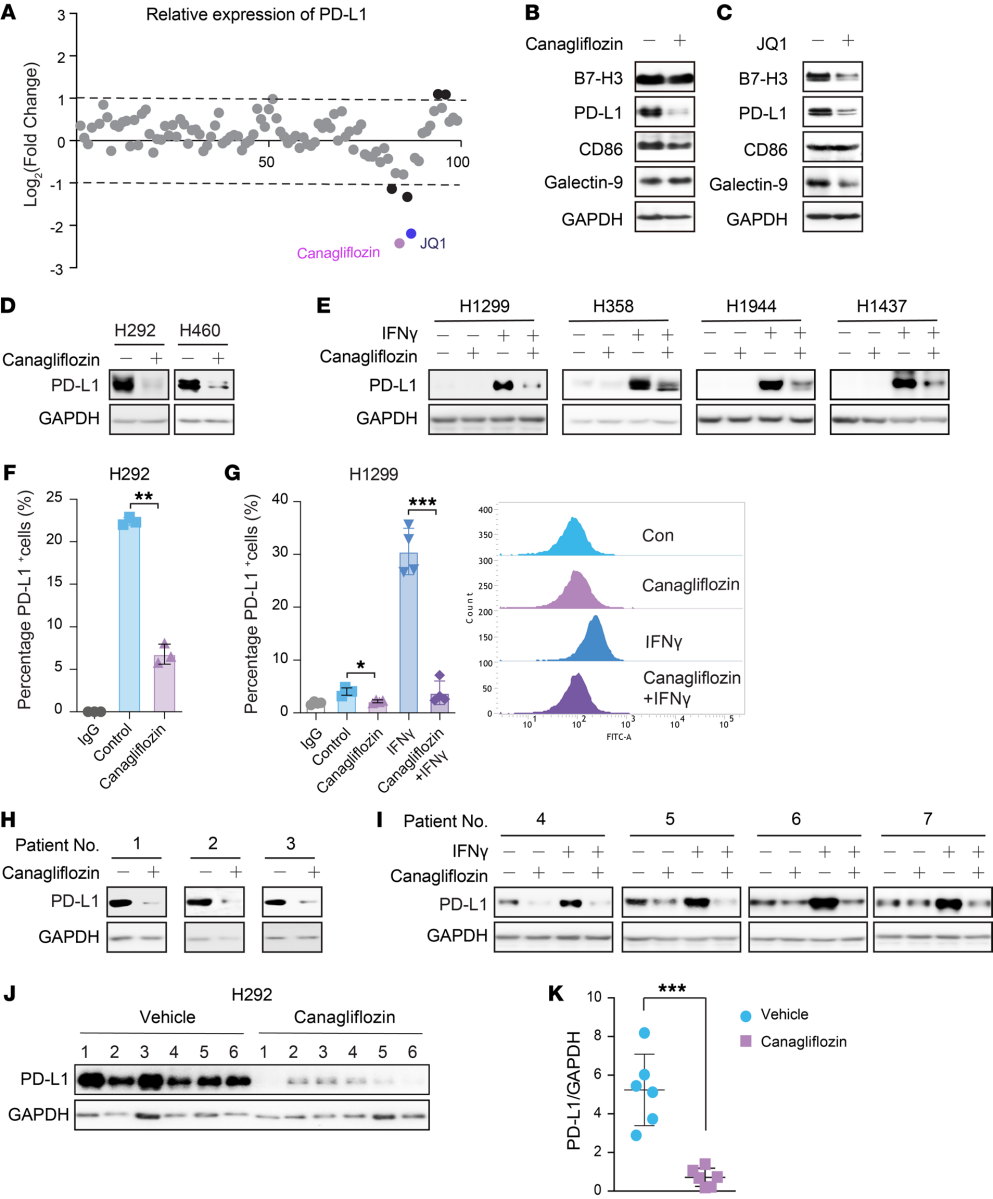
Canagliflozin suppresses PD-L1 expression in vitro and in vivo. (**A**) The effect of various small-molecule drugs on PD-L1 expression. H292 cells were treated with a compound library containing 98 small-molecule drugs (approved by the FDA) for 24 hours, followed by Western blot analysis with PD-L1 antibody and quantification using ImageJ grayscale analysis. JQ1 was used as a positive control that significantly downregulated PD-L1 expression. (**B** and **C**) Western blots depicting the effect of canagliflozin and JQ1 on regulating different checkpoint protein expression, blots were run in parallel. (**D** and **E**) Western blots depicting canagliflozin-downregulated expression of PD-L1 under basal (**D**) and inducible conditions (**E**). NSCLC cell lines H292, H460, H1299, H358, H1944 and H1437 were treated with canagliflozin (20 μM) alone or together with IFN-γ (10 ng/mL) for 24 hours, followed by detection of PD-L1 protein level by Western blotting. (**F** and **G**) Canagliflozin downregulated the expression of PD-L1 on the cell surface. Cell surface PD-L1 levels were investigated by flow cytometry in H292 (**F**) and H1299 (**G**) cells. Data were presented as the mean ± SD of triplicate (H292) or quadruplicate (H1299) experiments. IgG, Isotype control antibody control. (**H** and **I**) 7 cases of patient–derived primary NSCLC cancer cells were subjected to Western blotting analysis for PD-L1 expression after treatment with canagliflozin (20 μM) alone or together with IFN-γ (10 ng/mL) for 24 hours. (**J** and **K**) H292-implanted NSG mouse model was treated daily with canagliflozin (50 mg/kg body weight, intragastric administration) or vehicle for 1 week. Protein lysates from tumors were analyzed via Western blot and quantified using Image J grayscale analysis. *n* = 6 mice per experimental group. Blue circles, vehicle group; purple squares, canagliflozin group. Data were analyzed via unpaired 2-tailed Students’ *t* test. *P < 0.05; **P < 0.01; ***P < 0.001.

To identify small-molecule drugs that suppress PD-L1 expression, we performed a cell-based screening in a NSCLC cell line H292, which displays relatively high level of endogenous PD-L1. In our screening, 98 small-molecule drugs that have been approved for clinical use were included (Supplemental Table 1; supplemental material available online with this article; https://doi.org/10.1172/JCI154754DS1). In line with previous reports, a potent inhibitor of the BET family of bromodomain proteins, JQ1 (11), significantly suppressed PD-L1 expression (Figure 1A), confirming the reliability of our screening model. Intriguingly, the SGLT2 inhibitor canagliflozin exhibited the most effective inhibition of PD-L1 expression among these drugs (Figure 1A). Of note is the fact that, unlike the well-known broad transcriptional regulator JQ1, canagliflozin significantly and specifically downregulated PD-L1 protein in H292 without affecting other immune checkpoints (B7-H3, CD86,and Galectin-9) (Figure 1, B and C). To validate this result, we extensively examined the effect of canagliflozin on PD-L1 expression under both basal and inducible conditions. Again, canagliflozin reduced both constitutive and IFN-γ–induced PD-L1 expression in various NSCLC cell lines (Figure 1, D and E). Consistently, the level of PD-L1 on the cellular surface was decreased by canagliflozin (Figure 1, F and G). This observation was also validated in 7 separate instances of primary NSCLC patient-derived cancer cells (Figure 1, H and I). We also examined our findings in other cancers, including ovarian cancer, pancreatic cancer cell lines, and patient-derived cancer cells, and similar results were obtained (Supplemental Figure 1A). Thus, our findings were not restricted to lung cancer but applied to more cancers with SGLT2 expression. To test whether canagliflozin may affect PD-L1 expression under more pharmacologically relevant conditions, H292 cells were inoculated into the NSG mice and canagliflozin (50 mg/kg, converted from clinical dosage) was orally administered daily. Consistent with our in vitro results, PD-L1 expression in tumors was significantly decreased after 1 week of canagliflozin treatment (Figure 1, J and K). Taken together, these data suggest that canagliflozin is a potent small molecule that suppresses PD-L1 expression.

**Figure 2:**
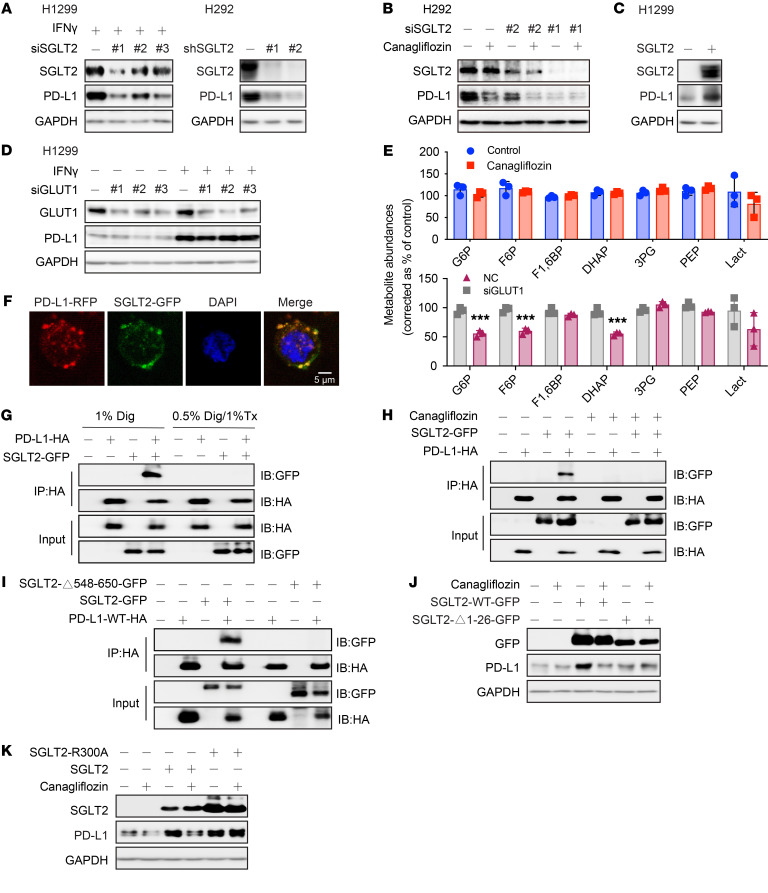
Canagliflozin reduces PD-L1 expression through its pharmacological target SGLT2. (**A**) Western blots showing that depletion of SGLT2 induced PD-L1 degradation. H1299 cells were treated with siRNAs targeting SGLT2. H292 cells were treated with shRNAs targeting SGLT2 as indicated. (**B**) Canagliflozin-caused PD-L1 decrease was abolished in the absence of SGLT2. (**C**) Overexpression of SGLT2 upregulated PD-L1 expression. (**D**) Depletion of GLUT1 had no effect on PD-L1 expression. H1299 cells were treated with siRNA-GLUT1 and the level of PD-L1 was detected by Western blotting. (**E**) Canagliflozin did not influence the abundance of glycolytic metabolites, whereas silencing of GLUT1 significantly reduced the abundance of glycolytic metabolites (*n* = 3). (**F**) Confocal analysis revealed the colocalization of SGLT2 and PD-L1 proteins in H1299 cells. Scale bar: 5 μm. (**G**) Interaction of SGLT2 with PD-L1 was detergent-sensitive. SGLT2-GFP and PD-L1-HA were transfected into HEK 293T cells for 24 hours. Cells were then lysed in 1% Digitonin (Dig) or 0.5% Digitonin/ 1% Triton X-100 (Tx) and immunoprecipitated with the anti-HA, followed by analysis using anti-GFP antibody. (**H**) Canagliflozin disrupted the interaction between SGLT2 and PD-L1. (**I**) Intracellular domain (aa 548–650) of SGLT2 was responsible for its interaction with PD-L1. (**J**) Downregulation of PD-L1 caused by canagliflozin was abolished when SGLT2 lost its plasma–membrane targeting region. H292 cells were treated with canagliflozin for 24 hours after transfection with SGLT2-GFP or SGLT2-Δ1-26-GFP. (**K**) Downregulation of PD-L1 caused by canagliflozin was abolished when the SGLT2 sodium-binding site was mutated. H292 cells were treated with canagliflozin for 24 hours after transfection with SGLT2 or SGLT2-R300A plasmids. Data were presented as the mean ± SD of triplicate experiments. Statistical significance was determined by unpaired 2-tailed Students’ *t* test. ***P < 0.001.

Given that SGLT2 is the pharmacological target of canagliflozin (14), we next investigated whether canagliflozin-mediated inhibition of PD-L1 is an on-target effect. We first knocked down the intracellular SGLT2 expression and found that PD-L1 was greatly decreased (Figure 2A). Canagliflozin–induced PD-L1 decrease was abolished in the absence of SGLT2 (Figure 2B). On the contrary, overexpression of SGLT2 upregulated the level of PD-L1 (Figure 2C). To test the specificity of PD-L1 regulation by SGLT2, we also included sodium-glucose cotransporter-1 (SGLT1) as a control. Our result showed that PD-L1 expression was not affected by depletion of SGLT1 (Supplemental Figure 2A). In addition to glucose transporter type 1 (GLUT1), which is mainly responsible for cellular glucose uptake, SGLT1 and SGLT2 are also expressed in various tumors and provide another effective way to increase glucose level in tumor cells (15). To determine whether the effect of SGLT2 on PD-L1 expression is due to the change in glucose uptake or glycolysis, we silenced GLUT1 by siRNA and found that expression of PD-L1 was not substantially altered upon depletion of GLUT1 (Figure 2D), while the abundance of glycolytic metabolites and glucose uptake (Figure 2E and Supplemental Figure 2B) were significantly decreased. On the contrary, canagliflozin at the concentration that we used — 20 μM — did not influence glucose uptake (Supplemental Figure 2B) or glycolysis (Figure 2E). Further, other SGLT2 inhibitors including LX-4211 and dapagliflozin also showed a suppressive effect on PD-L1 expression (Supplemental Figure 2, C and D). These findings clearly suggest that canagliflozin induced PD-L1 protein degradation through SGLT2 independent of its hypoglycemic effects.

Considering that SGLT2 is a transmembrane protein with 14 transmembrane helices, we tried to determine whether SGLT2 physically interacts with PD-L1. By confocal imaging analysis we confirmed that SGLT2 and PD-L1 colocalized on the cell membrane (Figure 2F and Supplemental Figure 2E). Next, we conducted coimmunoprecipitation assays using different detergent conditions to solubilize membrane proteins to variable degrees. SGLT2 was readily detected in association with PD-L1 only under conditions that preserve the integrity of a membrane-associated complex (Figure 2G). Notably, the interaction between SGLT2 and PD-L1 was disrupted by treatment of canagliflozin (Figure 2H). We thus tried to identify the binding region of SGLT2 with PD-L1. Enlightened by the differential effects of SGLT1 and SGLT2 on PD-L1 expression, after comparing the amino acid sequence of SGLT1 and SGLT2, we deleted the predicted intracellular domain of SGLT2 (aa 548–650). As expected, the physical interaction between SGLT2 and PD-L1 was abolished upon truncation of SGLT2 intracellular domain (Figure 2I).

Next, we tried to confirm whether the membrane localization of SGLT2 was required for its role in regulating PD-L1 stability. As shown by immunofluorescence assay, the SGLT2-GFP fusion protein missing residues 1–26 (SGLT2-Δ1-26-GFP) (16) lost the ability to localize at the plasma membrane, compared to the full-length SGLT2-WT-GFP protein (Supplemental Figure 2F). More importantly, SGLT2-Δ1-26-GFP also failed to upregulate PD-L1 expression (Figure 2J). Given that canagliflozin has been proven to bind from the external surface in a Na^+^-bound state, we generated SGLT2 with a mutation in the sodium-binding site (SGLT2 R300A and S392A/393A) (17, 18). We observed that sodium-binding mutants with exogenous expression of SGLT2 were able to upregulate PD-L1 expression, whereas canagliflozin could not downregulate PD-L1 when the SGLT2 sodium-binding site was mutated (Figure 2K and Supplemental Figure 2G), suggesting that canagliflozin induced a decrease in PD-L1 as an on-target effect. Taken together, SGLT2 is a positive regulator of PD-L1, and the interaction between SGLT2 and PD-L1 on the cell membrane is required for maintaining PD-L1 protein level.

**Figure 3:**
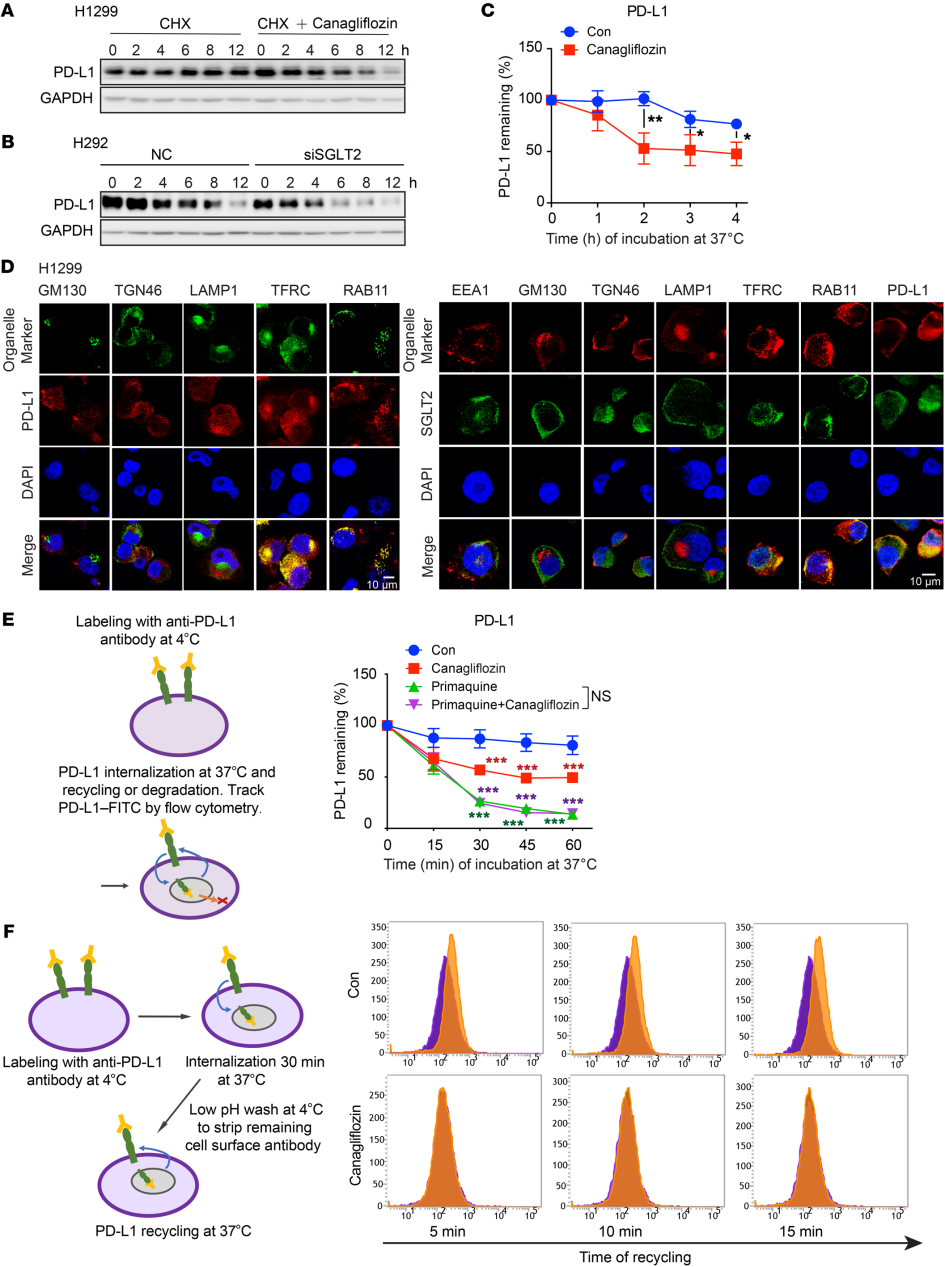
Canagliflozin inhibits the endocytic recycling of PD-L1. (**A**) Canagliflozin significantly attenuated the protein stability of PD-L1. H1299 cells were treated with IFN-γ (10 ng/mL) for 24 hours, then with cycloheximide (10 μg/mL), or cycloheximide (10 μg/mL) plus canagliflozin (20 μM) for the indicated time. (**B**) Depletion of SGLT2 promoted PD-L1 degradation. H292 cells were treated with siRNA-NC or siRNA-SGLT2 for 24 hours, followed by treatment with cycloheximide for indicated time. (**C**) Canagliflozin downregulated the expression of PD-L1 on cell surface (*n* = 3). (**D**) SGLT2 and PD-L1 colocalized with TFRC and RAB11. H1299 cells were fixed and costained with antibodies against SGLT2, PD-L1, and markers of Golgi (GM130 and TGN46), early endosome (EEA1), late endosome (LAMP1), or recycling endosome (RAB11 and TFRC). Scale bar: 10 μm. (**E**) Canagliflozin influenced the PD-L1 recycling process (*n* = 3). (**F**) Canagliflozin prevented internalized PD-L1 from recycling back to cell membrane. Purple shade represents the 0 minute group, and the orange shade represents 5, 10, and 15 minute groups. Data were presented as the mean ± SD (**C** and **E**). Statistical significance was determined by unpaired 2-tailed Students’ *t* test (**C** and **E**) and 1-way ANOVA with Dunnett’s post hoc test (**E**). *P < 0.05; **P < 0.01. ***P < 0.001.

To understand the mechanism of canagliflozin-induced PD-L1 suppression, we measured the mRNA level and protein half-life of PD-L1 in response to canagliflozin treatment. We found that canagliflozin significantly attenuated the protein stability of PD-L1 (Figure 3A and Supplemental Figure 3A), but not mRNA expression (Supplemental Figure 3B, C). Consistently, the half-life of PD-L1 was also reduced when SGLT2 was knocked down (Figure 3B). Also, the absence of SGLT2 caused a decrease in the half-life of PD-L1 that was reversed after reconstitution of SGLT2-WT. On the contrary, the reconstitution of SGLT2-Δ548-650 failed to prolong the stability of PD-L1 (Supplemental Figure 3D). These results further imply that canagliflozin accelerated PD-L1 protein degradation via the inhibition of SGLT2-mediated PD-L1 stability.

As the importance of endocytic recycling for maintaining PD-L1 protein stability has been recently revealed (19–21), we sought to explore whether canagliflozin induced PD-L1 degradation by interfering with this physiological process. PD-L1 on the cellular surface was labeled with PD-L1–specific antibodies at 4°C. Cells were then incubated in RPMI-1640 medium at 37°C to allow for PD-L1 internalization, degradation, or recycling to the cell membrane. As shown by flow cytometry assay, the degradation of labeled PD-L1 was significantly accelerated in the presence of canagliflozin (Figure 3C). Of note, canagliflozin did not cause PD-L1 degradation without internalization of PD-L1 in the presence of an endocytosis inhibitor (Supplemental Figure 3E). In addition, SGLT2 silencing also promoted the degradation of PD-L1 by applying this assay system (Supplemental Figure 3F). Meanwhile, cell surface MHC class I labeled under the same condition was similar between 2 groups, which was served as a system control here (Supplemental Figure 3G).

Encouraged by the facts that (a) PD-L1 was identified in recycling endosomes where it colocalized with TFRC and RAB11 — molecules that define endocytic recycling compartment (Figure 3D) — and (b) subcellular localization of SGLT2 was also identified in recycling endosomes (Figure 3D), we further hypothesized that SGLT2 might be pivotal for PD-L1 trafficking from recycling endosomes to the plasma membrane. To prove this, we first used primaquine, an endocytic recycling inhibitor, to prevent PD-L1 recycling to the plasma membrane. As expected, primaquine induced rapid loss of cell surface PD-L1, suggesting that a large proportion of surface PD-L1 is continuously internalized and recycled (Figure 3E). Of note, canagliflozin did not cause additional PD-L1 loss or acceleration of PD-L1 degradation in the presence of primaquine, which supports our hypothesis (Figure 3E). We next used the established flow cytometry–based recycling assay with some modification to estimate the portion of PD-L1 proteins recycled back to the plasma membrane (20), with or without the presence of canagliflozin. In principle, cell surface PD-L1 was labeled with FITC-conjugated PD-L1–specific antibody, which was allowed to internalize for 30 minutes at 37°C. The remaining cell-surface bound antibody was stripped by washing with pH 2.5 buffer and cells were either kept on ice (this was the poststrip baseline) or reincubated at 37°C for the indicated times. Recycled PD-L1 was analyzed by flow cytometry. As shown in Figure 3F, in control cells, the majority of internalized PD-L1 was recycled back to the cell surface after 10–15 minutes. However, PD-L1 recycling was markedly impaired by canagliflozin treatment. Taken together, canagliflozin specifically downregulated PD-L1 expression on the cell membrane by preventing the recycling of internalized PD-L1.

**Figure 4:**
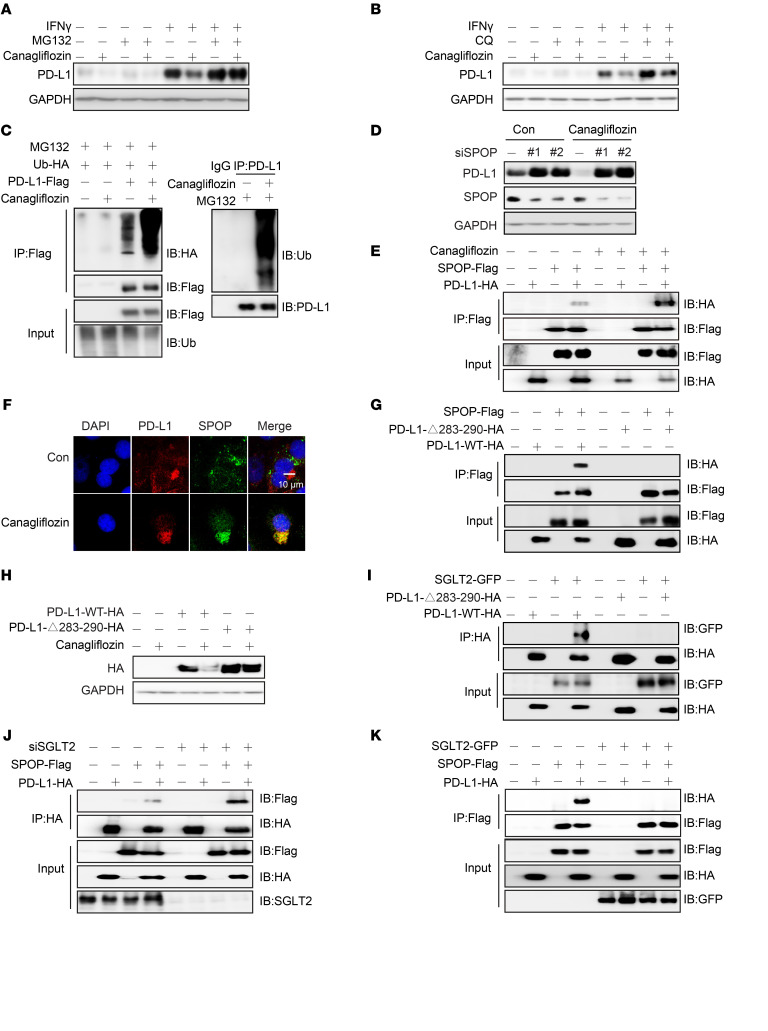
Canagliflozin induces PD-L1 degradation through the enhanced recognition of PD-L1 by Cullin3^SPOP^ ligase. (**A** and **B**) Canagliflozin degraded PD-L1 through the ubiquitin-proteasome pathway. H1299 cells were treated with canagliflozin with and without MG132 (**A**) or chloroquine (CQ) (**B**) for 10 hours. (**C**) Canagliflozin induced PD-L1 ubiquitination. Left, HEK 293T cells were transfected with indicated plasmids and were treated with canagliflozin and MG132. PD-L1 protein was immunoprecipitated with anti-Flag beads. Right, H1299 cells were treated with canagliflozin and MG132. PD-L1 protein was immunoprecipitated with PD-L1 antibody. (**D**) Canagliflozin failed to decrease PD-L1 expression upon SPOP silencing. PD-L1 protein expression in H460 cells was analyzed after treatment with canagliflozin in the presence of siRNAs against SPOP or negative control (siRNA-NC), blots were run in parallel. (**E**) Canagliflozin enhanced the interaction of SPOP and PD-L1. HEK 293T cells were treated with canagliflozin for 24 hours after transfection with SPOP-Flag or PD-L1-HA. The cell lysates were immunoprecipitated with anti-Flag resins. (**F**) Canagliflozin enhanced the colocalization of SPOP and PD-L1. H292 cells were treated with canagliflozin and the localization of SPOP and PD-L1 were detected by Immunofluorescence. Scale bar: 10 μm. (**G**) The intracellular domain of PD-L1 (aa 283–290) was responsible for the binding of PD-L1 to SPOP. HEK 293T cells were cotransfected with plasmids as indicated, cell lysates were immunoprecipitated with anti-Flag resins. (**H**) Downregulation of PD-L1 caused by canagliflozin was abolished upon deletion of the SPOP binding region. H292 cells were first transfected with PD-L1-WT-HA or PD-L1-283-290-HA, and then treated with canagliflozin. (**I**) SGLT2 bound to the same region of PD-L1 binding to SPOP (aa 283–290). HEK 293T cells were cotransfected with plasmid as indicated. The cell lysates were immunoprecipitated with anti-HA resins. (**J** and **K**) SGLT2 regulated the interaction between SPOP and PD-L1. SGLT2 was silenced (**J**) or overexpressed (**K**), and the interaction between SPOP and PD-L1 was subsequently determined.

Since both lysosome-dependent and proteasome-dependent degradation have been found to contribute to PD-L1 degradation during its endocytic recycling process, we sought to determine which pathway was involved in canagliflozin–triggered PD-L1 degradation. Coincubation of selective proteasome inhibitor MG132 abolished the downregulation of PD-L1 by canagliflozin (Figure 4A and Supplemental Figure 4A), whereas the specific lysosomal inhibitor chloroquine displayed no effect on PD-L1 (Figure 4B and Supplemental Figure 4B). Consistently, we also found that the level of ubiquitination of PD-L1 was significantly increased in the presence of canagliflozin (Figure 4C).

Next, we investigated which cullin family E3 ligase(s) might be involved in the process of PD-L1 ubiquitination. E3 ubiquitin ligases STUB1, β-TRCP, and SPOP (22), which have been reported to regulate PD-L1 ubiquitination, were knocked down. Interestingly, PD-L1 degradation caused by canagliflozin was not affected by STUB1 or β-TRCP deletion (Supplemental Figure 4, C and D), but was prevented by SPOP silencing (Figure 4D). Furthermore, coimmunoprecipitation and immunofluorescence assays suggested that canagliflozin enhanced the binding and colocalization between PD-L1 and SPOP (Figure 4, E and F). Previous studies have suggested that the region of the last 8 amino acids (aa 283–290) of PD-L1 represents the potential binding motif for SPOP, and our data also confirmed that PD-L1/SPOP interaction was diminished when this sequence was deleted (PD-L1-Δ283-290-HA) (Figure 4G). Importantly, the downregulating effect of canagliflozin was also abolished when PD-L1 lost its ability to interact with SPOP (Figure 4H). Together, these results further suggest that canagliflozin promoted PD-L1 degradation in a SPOP-dictated ubiquitination-dependent manner.

In addition, our data showed that the PD-L1 mutant with the deletion of the SPOP binding motif, PD-L1-Δ283-290-HA, also failed to bind with SGLT2 (Figure 4I). Thus, we asked whether the interactions of PD-L1/SGLT2 and PD-L1/SPOP compete with each other. To address this, we manipulated the intracellular expression of SGLT2 and examined its effect on the PD-L1/SPOP interaction. As shown in Figure 4, J and K, depletion of SGLT2 increased the binding of PD-L1 to SPOP, whereas overexpression of SGLT2 had the opposite effect. Collectively, these results demonstrated that canagliflozin-induced degradation of PD-L1 is mediated by SPOP, and SGLT2 plays a competitive role in regulating the interaction between PD-L1 and SPOP.

**Figure 5:**
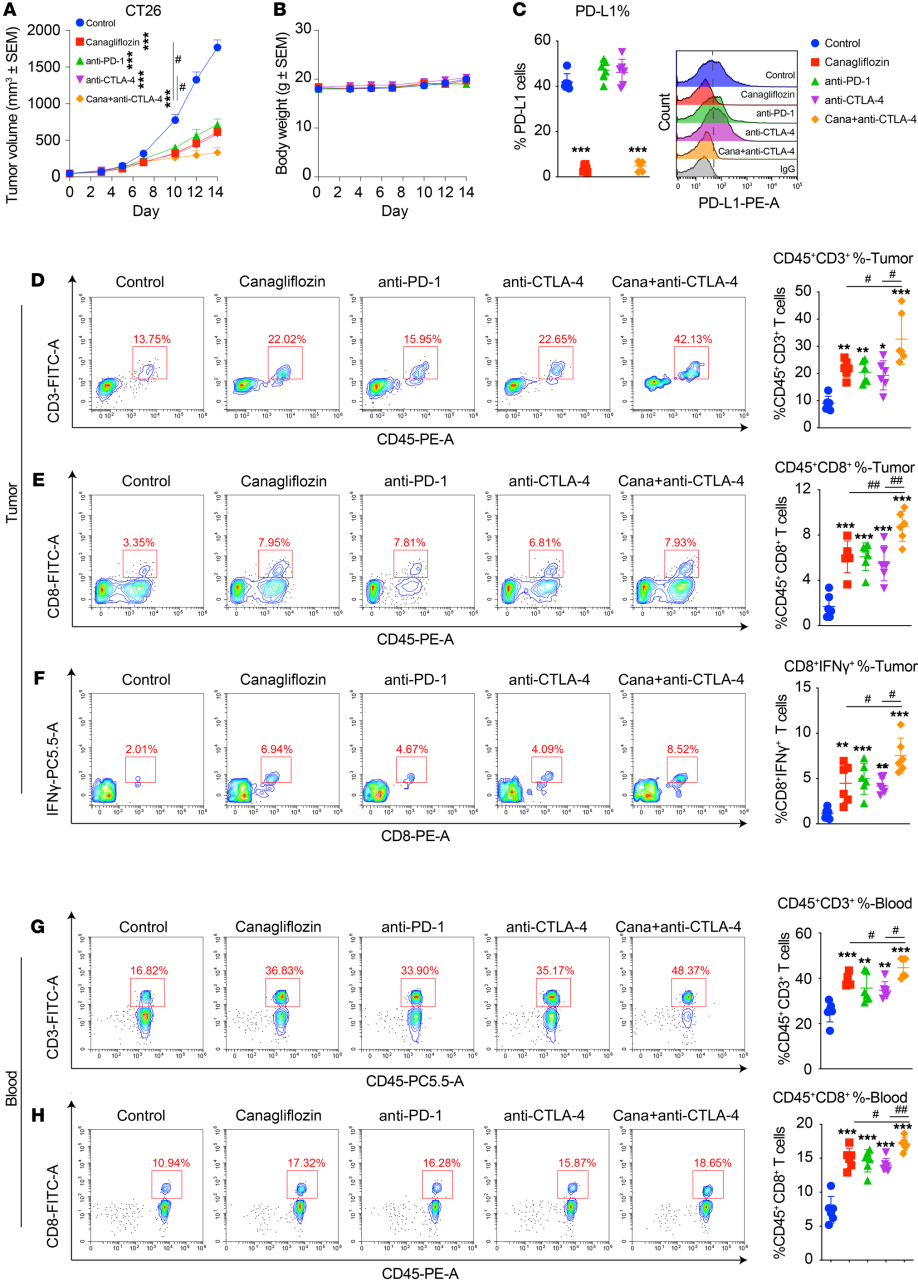
Canagliflozin alone, or combined with CTLA4 blockade, effectively suppressed tumor growth. (**A** and **B**) Tumor growth, weight, and volume of CT26 cells in immunocompetent BALB/c mice treated with canagliflozin, anti-PD-1 mAb, anti-CTLA4 mAb, or a combination of canagliflozin and anti-CTLA4 mAb. *n* = 6 mice per group. (**C**) PD-L1 level in extracted tumor tissues was evaluated by FACS, data represent mean ± SD. (**D** and **E**) Tumor-infiltrating CD45^+^CD3^+^ T cells and CD45^+^CD8^+^ T cells were detected by FACS, data represent mean ± SD. (**F**) FACS analysis of the activity intracellular IFN-γ in leukocytes, data represent mean ± SD. (**G** and **H**) CD45^+^CD3^+^ T cells and CD45^+^CD8^+^ T cells in blood were detected by FACS. Data represent mean ± SD. Data were analyzed by 1-way ANOVA with Dunnett’s post hoc test. *P < 0.05; **P < 0.01; ***P < 0.001; and unpaired 2-tailed Students’ t test ^#^*P* < 0.05; ^##^*P* < 0.01.

We next tried to determine whether canagliflozin could limit tumor growth in vivo using the CT26 mouse cancer model (23, 24). The CT26 mouse model is an immunocompetent mouse model with efficient PD-1/PD-L1 interaction (25), which is responsive to PD-L1 blockade and is widely used for PD-1 or PD-L1–based studies (26–28). Canagliflozin significantly suppressed tumor growth without affecting body weight, which was equivalent to the effect of anti-PD-1 antibody (Figure 5, A and B). In the tumors, PD-L1 level greatly decreased (Figure 5C), while tumor infiltrating CD3^+^ T cells, activated CD8^+^ T cells (Figure 5, D and E), and IFN-γ production increased significantly (Figure 5F). Similar observations were also found in blood (Figure 5, G and H). In line with clinical observations that the combination of anti-PD-L1 and anti-CTLA-4 antibodies achieved better therapeutic efficacy, we further found that cotreatment of canagliflozin and anti-CTLA-4 antibody significantly reduced the size of tumors compared with treatment with either canagliflozin alone or anti-CTLA-4 monotherapy (Figure 5A) without inducing substantial changes in body weight (Figure 5B). Consequentially, in the combination group, the level of activated CD8^+^ T cells and IFN-γ production were further increased in blood and tumor mass (Figure 5, D–H). Also, we compared the efficiency between anti-CTLA4 + anti-PD-L1 and anti-CTLA4 + canagliflozin groups. The combination of anti-PD-L1 and anti-CTLA4 antibodies achieved a comparable therapeutic efficacy compared with the anti-CTLA4 + canagliflozin group (Supplemental Figure 5, A and B)

**Figure 6:**
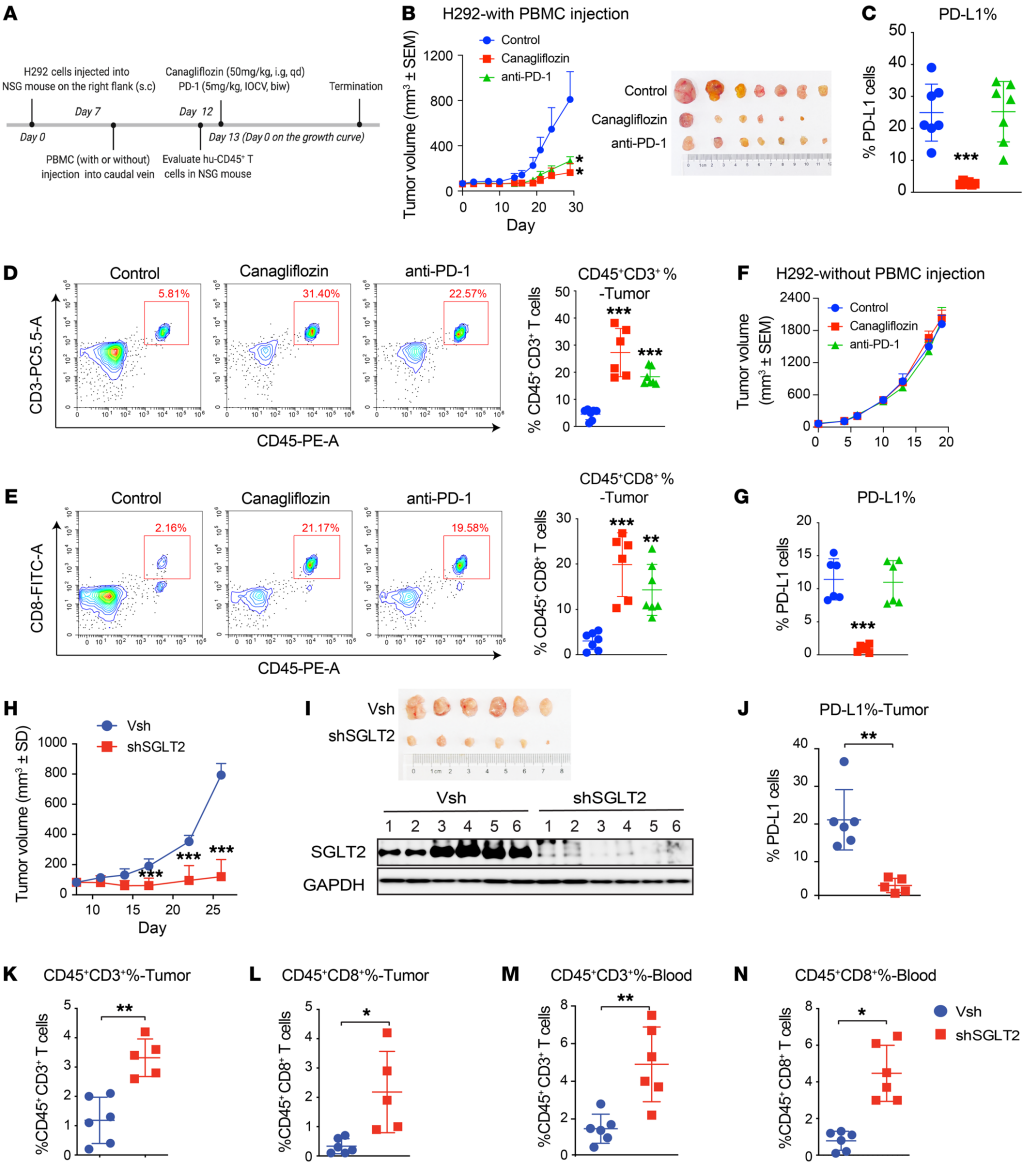
Canagliflozin effectively inhibits tumor growth in a PBMC humanized xenograft model. (**A**) Scheme representing the experimental procedure. s.c, subcutaneous; qd, 1 a day; i.g., intragastric; IOCV, injection of caudal vein; biw, twice per week. (**B**) Tumor growth of H292 cells in PBMC humanized NSG mice treated with vehicle, canagliflozin, or anti-PD-1 Ab. *n* = 7 mice per group. (**C**) PD-L1 level in extracted tumor cells was evaluated by FACS. (**D** and **E**) Tumor-infiltrating CD45^+^CD3^+^ T cells and CD45^+^CD8^+^ T cells were detected by FACS. (**F**) Tumor growth of H292 cells in immuno-deficient NSG mice when treated with vehicle, canagliflozin, or anti-PD-1 Ab. *n* = 6 mice per group. (**G**) PD-L1 level in extracted tumor cells from immuno-deficient NSG mice was evaluated by FACS. (**H** and **I**) shSGLT2 significantly inhibited the tumor growth in the humanized NSG mouse model. H292 cells with or without SGLT2 knocked down were injected into PBMC humanized NSG mice and tumor growth was measured. *n* = 6 mice per group. (**J**) The surface level of PD-L1 on tumor cells were evaluated by FACS. (**K** and **L**) Tumor infiltrating CD45^+^CD3^+^ T cells and CD45^+^CD8^+^ T cells were detected by FACS. (**M** and **N**) CD45^+^CD3^+^ T cells and CD45^+^CD8^+^ T cells in blood were detected by FACS. Data represent mean ± SD. Statistical significance was determined by 1-way ANOVA with Dunnett’s post hoc test (**B**–**E** and **G**) and unpaired 2-tailed Students’ *t* test (**H** and **J**–**N**). *P < 0.05; **P < 0.01; ***P < 0.001.

To confirm that canagliflozin suppressed tumor growth mainly through the suppression of PD-L1 and the existing immune system, we performed a similar study in PBMCs engrafted into a humanized xenograft model. The injection of human PBMCs into immunodeficient mice, also known as the Hu-PBL-SCID model, leads to the engraftment of T cells, which provides a unique platform in which the tumor microenvironment (TME) can be evaluated in vivo. This model may also be beneficial in the assessment of immune checkpoint inhibitors, mimicking the interaction between immune cells and tumor cells in vivo (28–30). H292 cells were inoculated subcutaneously in NOD-*Prkdc*^scid^*Il2rg*^em1^/Smoc (NSG) mice humanized with PBMCs (Figure 6A). Tumor growth was monitored when the mice were treated with canagliflozin or anti-PD-1 mAb. In models humanized by the addition of PBMCs, canagliflozin significantly suppressed tumor growth and such effect was similar to anti-PD-1 antibody treatment (5 mg/kg, twice a week) (Figure 6B). Moreover, canagliflozin decreased PD-L1^+^ cells present in the tumor mass (Figure 6C). The population of tumor-infiltrated CD3^+^ T cells and activated CD8^+^ T cells was increased in canagliflozin-treated mice, which resembled the anti-PD-1 antibody treated tumor (Figure 6, D and E). On the contrary, we found that without the infusion of PBMCs, no antitumor effect was observed in the canagliflozin group or the anti-PD-1 antibody group (Figure 6F); even canagliflozin exerts a significant inhibitory effect on PD-L1(Figure 6G). Further, we constructed PD-L1–KO tumor cells by CRISPR/Cas9 and established an in vivo model to evaluate the antitumor effect of canagliflozin. In both the CT26 and H1299 models, the antitumor effect of canagliflozin was abolished when PD-L1 was knocked out by CRISPR/Cas9-mediated genome editing (Supplemental Figure 5, C–F). These data further suggested that the canagliflozin induced antitumor effect is dependent on the presence of PD-L1.

Similar results were also achieved by knockdown of SGLT2 in H292 cells. Although the cell proliferation of H292 was not significantly affected when SGLT2 was absent (Supplemental Figure 6A), shRNA targeting SGLT2 (shSGLT2) significantly inhibited tumor volume compared with the control group (Vsh group) in the humanized NSG mouse model (Figure 6H). In parallel, the silencing efficacy of shSGLT2 was verified (Figure 6I). Flowcytometric analysis of tumor tissues showed that shSGLT2 significantly reduced the expression of PD-L1 (Figure 6J) and boosted the population of activated tumor-infiltrated CD3^+^ and CD8^+^ T cells (Figure 6, K and L). Because the tumor of 1 mouse in the shSGLT2 group was too small for flow cytometry, data was collected from 6 Vsh and 5 shSGLT2 mice. CD3^+^ T cell and activated CD8^+^ T cell populations were also increased in the blood in the shSGLT2 group (Figure 6, M and N). On the contrary, in the NSG mouse model without PBMC injection, tumor growth was not significantly affected by SGLT2 knockdown (Supplemental Figure 6B). These observations further indicated that SGLT2–mediated tumor growth depended on the existing immune system. The above data suggest that SGLT2 played an important role in maintaining the level of PD-L1 in tumor cells, and that the intervention of SGLT2 can induce antitumor effects through immune regulation.

**Figure 7:**
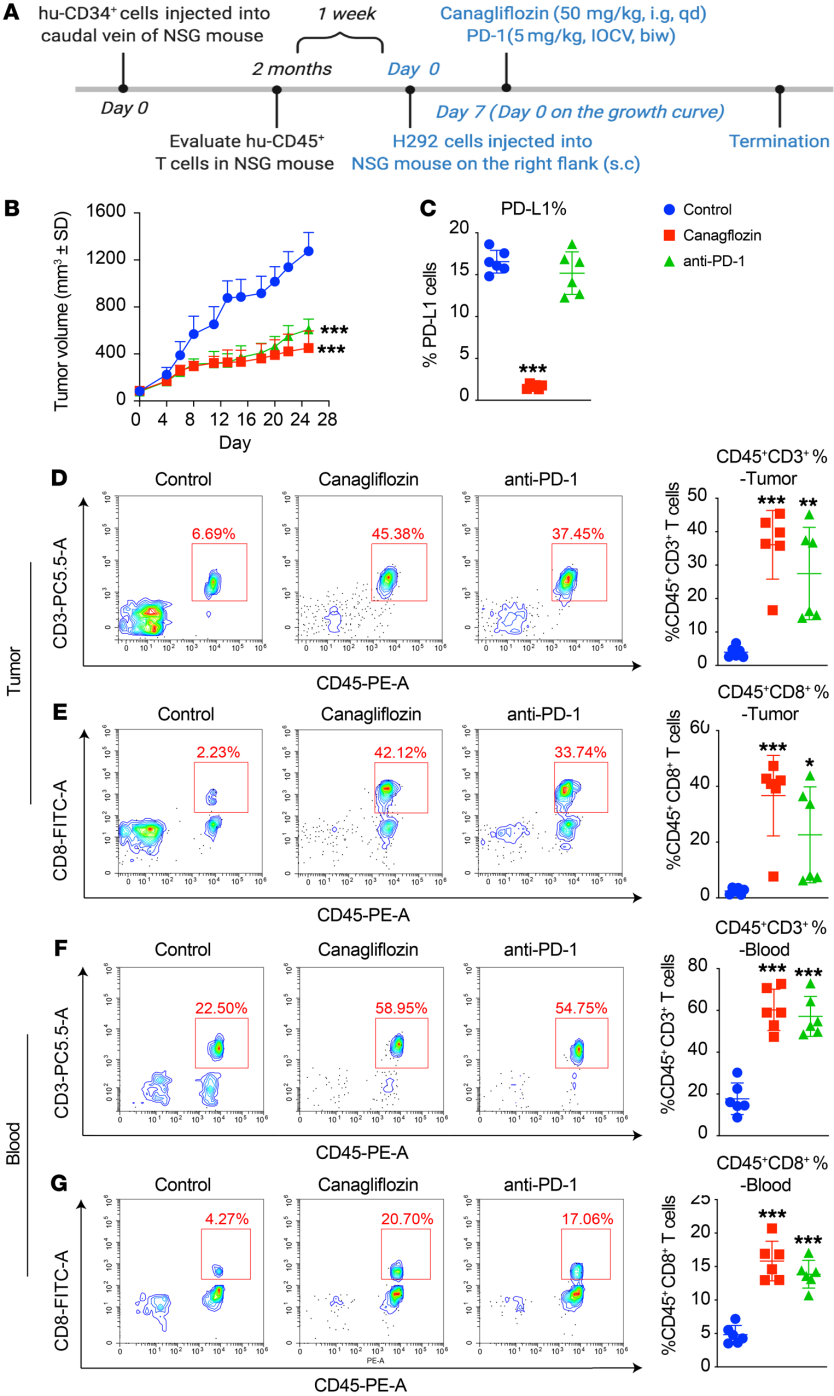
Canagliflozin effectively suppresses tumor growth in CD34^+^ stem cells engrafted into a humanized xenograft model. (**A**) Scheme representing the experimental procedure. s.c, subcutaneous; qd, 1 a day; i.g, intragastric; IOCV, injection of caudal vein. (**B**) In humanized immune-transformed model, H292 cells were injected subcutaneously and treated with vehicle, canagliflozin, or anti-PD-1 Ab. *n* = 6 mice per group. (**C**) PD-L1 levels on extracted tumor cells were evaluated by FACS. (**D** and **E**) Tumor infiltrating CD45^+^CD3^+^ T cells, CD45^+^CD8^+^ T cells were detected by FACS, data represent mean ± SD. (**F** and **G**) CD45^+^CD3^+^ T cells and CD45^+^CD8^+^ T cells in blood were detected by FACS. Data represent mean ± SD. Statistical significance was determined by 1-way ANOVA with Dunnett’s post hoc test. *P < 0.05; **P < 0.01; ***P < 0.001.

To better mimic the human cancer environment, NSG mice were humanized by adoptive transfer using human umbilical cord blood–derived CD34^+^ stem cells from a qualified source, following myeloablation treatment. The injection of CD34^+^ stem cells into newborn or young mice, also known as the Hu-SRC-SCID model, allows for the differentiation and development of a more complete immune system, including T cells, B cells, and innate immune cells; this model provided a more physiologically relevant tumor microenvironment, mimicking the interaction between immune cells and tumor cells in vivo (31, 32). Successful humanization of each mouse is quantified from mouse peripheral blood via flow cytometry using anti-hu-CD45^+^ and anti-murine-CD45^+^ antibodies approximately 2 months after engraftment, when mature T cells develop. Mice included in the study have more than 15% hu-CD45^+^ cells in the peripheral blood. Mice were then treated with canagliflozin and PD-1 antibody as indicated (Figure 7A). Canagliflozin treatment reduced tumor size, showing effects similar to the anti-PD-1 antibody (Figure 7B). In agreement with our proposed mechanism, flowcytometric analysis of tumor tissues showed that canagliflozin reduced PD-L1 levels and increased the CD3^+^ T cell and CD8^+^ T cell populations (Figure 7, C–E). Similar observations were also found in blood (Figure 7, F and G). Consistently, these in vivo data demonstrated the antitumor immunity of canagliflozin and its potential application in cancer therapy.

**Figure 8:**
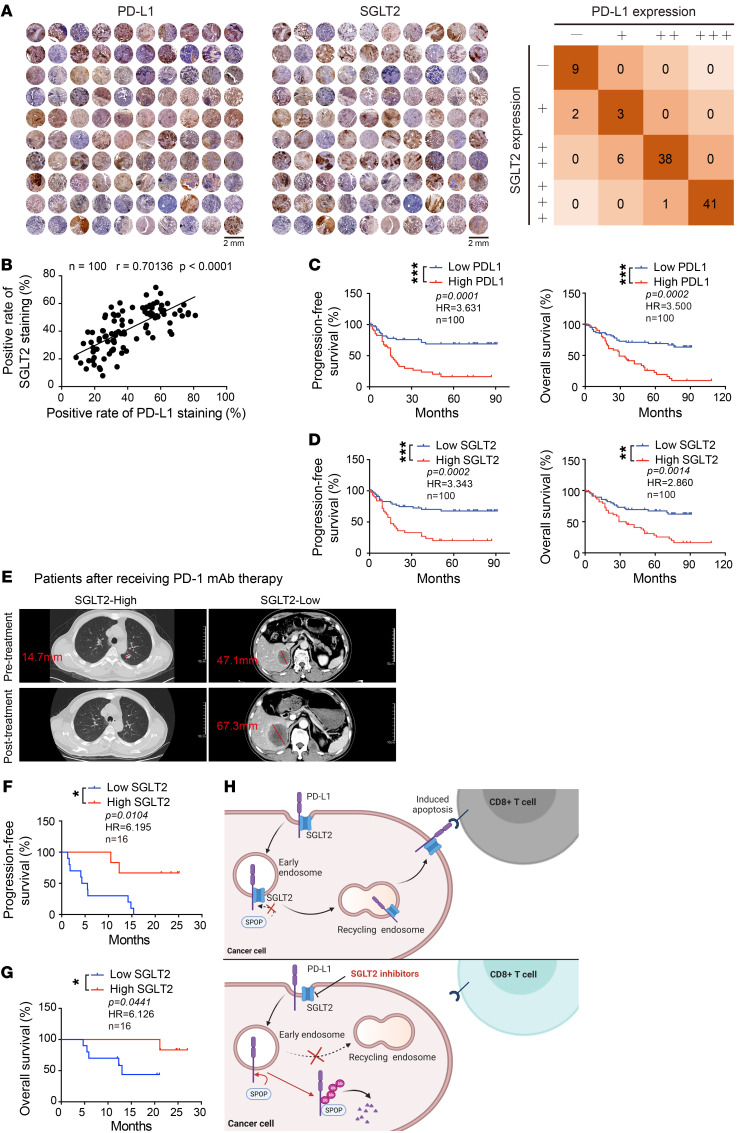
SGLT2 positively correlated with PD-L1 expression in lung cancer tissues. (**A** and **B**) Patient tissues were stained with SGLT2 and PD-L1. Representative images of IHC staining of SGLT2 and PD-L1 in human lung cancer tissues (*n* = 100) were shown. Scale bar: 2 mm. The correlation analysis between SGLT2 and PD-L1 was performed, and the *P* value was calculated by the Pearson correlation test (*P* < 0.0001, r = 0.70136). –, negative expression; +, low expression; ++, medium expression; +++, high positive expression. (**C** and **D**) Kaplan-Meier survival curves of NSCLC patients’ PFS or OS. The low expression category includes those whose positive staining rate is smaller than 50%, whereas the high expression category greater than 50%. The Gehan-Breslow-Wilcoxon test was used to test for the difference between survival curves. (**E**) Tumor diameter based on the CT imaging was annotated with a red line. Scale bar: 10 cm. (**F** and **G**) Kaplan-Meier survival curves of NSCLC patients’ PFS or OS. The Gehan-Breslow-Wilcoxon test was used to test for the difference between survival curves. See also Supplemental Tables 3–5. (**H**) Diagram of the mechanism of SGLT2 regulating PD-L1.*P < 0.05, **P < 0.01; ***P < 0.001.

To validate the above model in human tumor tissues, we assessed protein expression levels of SGLT2 and PD-L1 in a cohort of 100 patients with lung cancer (Figure 8A, Supplemental Figure 7A, and Supplemental Table 2). Pearson’s χ**^2^** test showed a positive correlation between SGLT2 and PD-L1 expression in specimens from patients with cancer (Figure 8B). Next, we evaluated the correlation between the SGLT2-PD-L1 axis and the prognosis of patients with lung cancer. As shown in Figure 8, C and D, we observed reduced progression-free survival (PFS) and overall survival (OS) curves in patients with lung cancer with high expression of SGLT2 and high expression of PD-L1, suggesting that SGLT2 could be utilized as a marker for the expression of PD-L1.

Moreover, we further assessed the protein expression level of SGLT2 by IHC in tumor biopsies from patients with NSCLC who were treated with PD-1 mAb therapy. Among a total of 16 patients recruited, 9 patients with a positive response to PD-1 mAb therapy were classified as responders, while the other 7 patients with a poor response were classified as nonresponders (patients with complete response [CR], partial response [PR], and stable disease [SD] greater than 6 months were classified as responders, while patients with SD less than or equal to 6 months and progressive disease [PD] were classified as non-responders; Supplemental Tables 3–5). Similar to the pathology results described above, radiographic results were shown to corroborate this correlation. Two representative cases with tumor diameter annotated by a radiologist were shown in Figure 8E (red line). Considering a cutoff point of 50% to demarcate presence or absence of SGLT2 expression level, patient 1 had greater than 50% SGLT2 expression and showed tumor destruction after anti-PD1 therapy (Figure 8E, left), while patient 2, with hepatic metastasis from lung cancer and less than 50% SGLT2 expression, showed tumor growth after treatment (Figure 8E, right). As shown in Figure 8, F and G, we observed prolonged PFS and OS curves in the group of PD-1 blockade-treated patients with high expression of SGLT2. This clinical evidence further suggests that SGLT2 is a positive regulator of PD-L1.

## Discussion

Increasing research attention has focused on understanding the regulatory mechanism of PD-L1 and the small molecule compounds that can modify PD-L1 expression (33). Our study identifies SGLT2 binding to PD-L1 at the plasma membrane and in recycling endosomes, where it protects PD-L1 from ubiquitination-mediated degradation. Canagliflozin, an SGLT2 inhibitor, disrupts the interaction between PD-L1 and SGLT2, and thereby enhances the interaction between PD-L1 and the E3 ligase SPOP (Figure 8H). Further, canagliflozin exhibits a significant antitumor effect in both syngeneic models and humanized immune-transformation models. Notably, various studies have uncovered the pathways modulating PD-L1 at epigenetic, transcriptional, translational, and posttranslational levels (22), and our findings define a mechanism for maintaining cell surface PD-L1 stability, raising the possibility of targeting PD-L1 by canagliflozin.

SGLT2 is a Na^+^-D-glucose cotransporter belonging to the *SLC5A* gene family, which harnesses the gradient of sodium ions across the plasma membrane to drive glucose and other nutrients into cells (15, 34). In humans, SGLT2 is mainly expressed in the kidney and specialized regions of the brain, while it is hardly detectable in other tissues (35, 36). Recent evidence has demonstrated the expression of SGLT2 in lung (37), pancreatic, and prostate cancer tissues (34), as well as the functional activity of this protein as a glucose transporter in cancer cells. However, in most tumors, GLUT1 is upregulated to meet the increasing demand for glucose (38), and the importance of SGLT2 in glucose uptake has not yet been clarified. Here, we identified SGLT2 as a master regulator of PD-L1 cell surface expression. SGLT2 knockdown leads to PD-L1 degradation during recycling, and recycling failure ultimately results in PD-L1 exhaustion. In this case, PD-L1 was still dynamically expressed and localized to the membrane, and endocytic recycling and degradation took place during tumor growth, which was why immune cells were stimulated in the animal study using SGLT2 shRNA (Figure 6, H, K, and L). However, immune cell stimulation was not observed in the PD-L1–knockout model (Supplemental Figure 5, C–F). In this case, tumors grew without PD-L1 due to another compensating mechanism. It is unclear whether tumors undergo formation or destruction in an immunosuppressive environment. Our findings indicate the involvements of SGLT2 in immune escape via stabilization of cell surface PD-L1.

Canagliflozin, which lowers glucose levels by inhibiting SGLT2, was developed to treat type 2 diabetes (14). Recent studies have shown that canagliflozin induces antitumor effects by reducing glucose uptake (37, 39). Here we report that canagliflozin triggers PD-L1 degradation and inhibits tumor growth in the presence of a functional immune system, suggesting that the antitumor effect of canagliflozin is linked to immune response. How canagliflozin disrupts the interaction between PD-L1 and SGLT2 was not clear in our study. As a transporter, SGLT2 isomerizes between conformations to shuttle cargo across membranes. SGLT2 inhibitors binding on the extracellular surface are likely to stabilize an outward-facing conformation (14, 40). Thus, we may infer that SGLT2 in an outward-facing conformation would likely dissociate from PD-L1. We also checked other SGLT2 inhibitors, LX-4211 and dapagliflozin — which is as potent as canagliflozin(41) — and found less suppressive effects on PD-L1 expression. This result suggests that canagliflozin is most potent in stabilizing SGLT2 in an outward-facing state. However, we could not provide direct evidence in the current study to support this proposal.

Recent studies highlight the potential to further enhance the clinical benefits of monotherapies by combining agents with synergistic mechanisms of action. The anti-PD-1 mAb and anti-CTLA-4 mAb combination has been recently reported to achieve better therapeutic efficacy in several clinical trials (42, 43). However, combined treatment of 2 antibodies further increases the risk of severe adverse reactions (44). Here we showed that canagliflozin and anti-CTLA-4 also synergistically activated T cells in a tumor xenograft model and retarded tumor growth. As a drug for diabetes, canagliflozin does not show significant adverse effects in clinical use (45). Because of this, we propose that canagliflozin has the advantage in combination therapy. Further, in clinical treatment for diabetes, the maximal dose of canagliflozin is 300 mg daily for an adult (46). Here, we showed that significant tumor growth limitation and PD-L1 suppression were achieved with treatment of 50 mg/kg canagliflozin, which was converted from the clinical dosage. Thus, our study provides evidence for the potential application of canagliflozin in immunotherapy.

Tumor PD-L1 expression is considered a potential efficacy biomarker (47), but the complex mechanism underlying its regulation is not completely elucidated. Tumor cells with constitutive PD-L1 expression are not typically linked to the response to immunotherapy when the T cell environment is absent (1). Therefore, PD-L1 expression alone is not a strong predictive biomarker of immune checkpoint blockade efficacy (48). It is crucial to explore the regulatory mechanism of PD-L1 expression in response to IFN-γ secreted by activated T cells and to discover novel theragnostic markers for PD-1/PD-L1-based therapies. Herein, we discovered that SGLT2 bound to PD-L1 on the cell surface and maintained the stability of PD-L1. Thus, SGLT2 may serve as a potential predictor for the efficacy of PD-1 mAb therapy.

In summary, we have identified SGLT2 as a PD-L1 binding partner, which prevents PD-L1 from proteasome-dependent degradation in endocytic recycling. Our finding could provide a ready-to-use small-molecule drug and a potential target for triggering PD-L1 degradation. Also, canagliflozin could be a good candidate for the development of combination therapy. Taken together, our study provides a view for the design of an effective strategy to target PD-L1 degradation in tumor immunotherapy.

## Methods

*Cell culture*. All the cell lines were provided by Cell Bank of Shanghai Institute for Biological Sciences, Chinese Academy of Sciences (Shanghai, China) and were authenticated by short tandem repeat DNA fingerprinting, with the most recent authentication on September 15, 2020. All cell lines were tested and verified to be free of Mycoplasma. NCI-H1299, NCI-H1437, NCI-H358, NCI-H1944, NCI-H460, NCI-H292, SKOV3, and CT26 were cultured in RPMI-1640 (Gibco) supplemented with 10% FBS (Hyclone). MIA PaCa-2, HEK293T, and HEK293FT were maintained in DMEM (Gibco) with 10% FBS (Gibco). All the cell lines were cultured in a humidified incubator at 37°C in 5% CO_2_.

*Primary cancer cell isolation*. The blood stasis and nontumor tissue on the surface of cancer tissues were cleaned twice with RPMI-1640, then cancer tissues were mechanically dissociated and cut into rice-grain-sized blocks with sterilized surgical instruments. The cancer blocks were maintained in DMEM/F-12 (Gibco) with 10% FBS. Subsequently, fresh culture solution was replaced every 2 days until dissociated cancer cells grew from the tissue. Cells were cultured in a humidified incubator at 37°C in a 5% CO_2_.

### Clinical tissue samples

Lung cancer, ovarian cancer, and pancreatic cancer tissues, paraffin sections from patients with lung cancer who responded or did not respond to Nivolumab (3 mg/kg, once every 2 weeks), and tissue microarrays from patients with lung cancer were collected from Zhejiang Cancer Hospital and written informed consent was obtained from clinical patients in all cases at the time of enrollment. Clinical information is summarized in Supplemental Tables 2–5.

### Antibodies and reagents

#### Immunofluorescence.

Mouse anti-PD-L1 (14-5983-82), mouse anti-EEA1 (14-9114-82), and mouse anti-LAMP1 (14-1079-80) were purchased from eBioscience. Mouse anti-GM130 (ab169276), mouse anti-TGN46 (ab2809), and rabbit anti-PD-L1 (EPFR19759) were obtained from Abcam. Rabbit anti-SGLT2 (24654-1-AP, Proteintech), mouse anti-TFRC (A-11130, Invitrogen), and mouse anti-Rab11 (610656, BD Biosciences) were also used.

### Flow cytometry

FITC mouse IgG1 κ isotype control (400110), PerCP-Cy5.5 mouse IgG1 κ isotype control (400149), PE mouse IgG1 κ isotype control (400114), PerCP-Cy5.5 anti-human CD3 (300430), FITC anti-human CD8a (300906), PE anti-human CD45 (368510), PerCP-Cy5.5 anti-human IFN-γ (506528), FITC Rat IgG2a κ isotype control (400506), PerCP-Cy5.5 Rat IgG1 κ isotype control (400426), FITC anti-mouse CD3 (100204), FITC anti-mouse CD8a (100705), PE anti-mouse CD45 (103106), and PerCP-Cy5.5 anti-mouse IFN-γ (505822) were purchased from Biolegend. PE anti-human PD-L1 (557924), FITC anti-human PD-L1 (558065), FITC anti-human HLA-A2 (343303), FITC Rat IgG2b κ isotype control (400605), PE Rat IgG2a λ isotype control (400635), and PE anti-mouse PD-L1 (558091) were obtained from BD Biosciences.

#### Immunoprecipitation/immunoblotting.

Mouse anti-Galectin-9 (ab153673), rabbit anti-GLUT1 (ab115730) were from Abacm. Rabbit anti-CD86 (91882), rabbit anti-B7-H3 (14058), rabbit anti-PD-L1 (13684), and rabbit anti-SGLT2 (14210) were purchased from Cell Signaling Technology. Rabbit anti-GAPDH (db106), rabbit anti-HA (db2603) were from Diagbio. Mouse anti-Ub (sc-8017), mouse anti-STUB1 (sc-133066), mouse anti-β-TRCP (sc-390629) were obtained from Santa Cruz Biotechnology. Rabbit anti-SPOP (16750-I-AP, Proteintech), and rabbit anti-Flag (A01868, GenScript) were also used.

#### IHC.

Mouse anti-PD-L1 (TA321380S, Origene) and rabbit anti-SGLT2 (ab85626, abcam) were used in IHC analysis.

#### Reagents.

The library of FDA-approved drugs, which contains 98 chemical compounds dissolved at 10 mM in dimethylsulfoxide; canagliflozin (T1782); LX-4211 (T3547); dapagliflozin (T2389); JQ1 (T2110); primaquine (T0850); chlorpromazine (T1384); MG132 (T2154); and chloroquine (T8689) were purchased from TargetMol. IFN-γ was purchased from PeproTech (300-02-1000). Sulforhodamine B (230162) and cycloheximide (#239763-M) were purchased from Sigma-Aldrich. 2-DG uptake Glucose Uptake Assay Kit (Colorimetric) (ab136955, abcam) was used in analysis.

### Western blotting and immunoprecipitation analyses

For Western blotting, cells were washed twice with cold PBS and lysed either in 1% NP-40 lysis buffer (25 mmol/L Tris-base, pH 7.4, 150 mmol/L NaCl, 10% glycerol) or in lysis buffer containing 25 mmol/L Tris-base (pH 7.4), 150 mmol/L NaCl, 1% NP-40, 1 mmol/L PMSF, 1 mmol/L Na_3_VO_4_, and 5 μg/mL leupeptin for 30 minutes on ice followed by the removal of insoluble material by centrifugation. Equal amounts of protein were loaded into SDS-PAGE gels for electrophoresis and immunoblotted with the indicated antibodies. See complete unedited blots in the supplemental material.

For immunoprecipitation of protein ubiquitination, cells were lysed in 4% SDS buffer (4% SDS, pH 8.0, 150 mmol/L NaCl, 50 mmol/L triethylamine), followed by sonication and centrifugation at room temperature and diluted 1:9 with 1% NP-40 lysis buffer. Cell lysates were immunoprecipitation by incubating with anti-Ub overnight at 4°C and then pulled down with 30 μL protein A/G beads (Santa Cruz Biotechnology) at 4°C for 1 hour to capture antibody-bound Ub. Samples were then loaded in SDS-PAGE gels and immunoblotted as described.

For coimmunoprecipitation experiments, HEK293T cells were lysed in lysis buffer (25 mmol/L Tris-base, pH 7.4, 150 mmol/L NaCl, 1% NP-40, 1 mmol/L PMSF, 1 mmol/L Na_3_VO_4_, and 5 μg/mL leupeptin). We used the BCA protein assay (Yeasen Biotech) to measure protein concentration. Lysates (1 mg) were incubated with anti-HA affinity gel or anti-DYKDDDDK (Flag) beads at 4°C overnight. Sample protein was then loaded in SDS-PAGE gels and immunoblotted as described. For coimmunoprecipitation of cell surface protein, cells were lysed in 1% Digitonin (20) (Millipore) (50 mmol/L Tris-HCl, pH 7.5, 150 mmol/L NaCl, 1% Digitonin, 1 mmol/L PMSF, 1 mmol/L Na_3_VO_4_, and 5 μg/mL leupeptin) to ensure the integrity of membrane proteins.

### Flow cytometry

Single-cell suspensions were washed with PBS and stained with FITC-conjugated PD-L1 at 4°C for 2 hours (5 μL / 2 × 10^5^ cells in 100 μL 0.2% BSA). After washing with PBS, samples were analyzed on BD FACSuite (BD Biosciences). Mouse tumor tissues were first separated into single cells using a tissue dissociator (Miltenyi Biotec). Single cells were stained with antibodies at room temperature for 30 minutes (1 μL / 2 × 10^5^ cells in 100 μL 0.2 % BSA) and then treated as described above. Mouse blood samples were incubated with antibodies at room temperature for 30 minutes (1 μL / 50 μL blood sample). Blood samples were lysed in red blood cell lysis buffer according to standard lysis buffer kit (BD Biosciences) protocol and flow cytometry analysis was performed as described above.

### Endocytosis and recycling assays

Endocytosis and recycling assays were performed based on methodology found in Burr et al. (20) and are described below.

#### Degradation assay.

Cells were collected and washed with PBS. Cell surface PD-L1 or MHC I was labelled with FITC-conjugated PD-L1 or MHC I at 4°C for 2 hours (5 μL / 2 × 10^5^ cells in 100 μL 0.2% BSA). Cells were washed in PBS to remove unbound antibodies and replated in RPMI-1640 medium to incubate at 37°C for indicated time in the presence or absence of canagliflozin (20 μM). Alternatively, cells were first transfected with Control shRNA or shSGLT2 for 72 hours, and then cells were collected and washed with PBS. Cells were labeled as described above with FITC-conjugated PD-L1 and washed in PBS to remove unbound antibodies, then replated in RPMI-1640 medium to incubate at 37°C. At the indicated times, cells were fixed with 4% paraformaldehyde for 20 minutes and removed in ice, washed in PBS, and analyzed by flow cytometry.

#### Internalization assay.

Cells were labeled as described above with FITC-conjugated PD-L1. After washing as described, cells were resuspended in RPMI-1640 medium, and baseline samples were kept on ice. Cells were cultured at 37°C in the presence or absence of canagliflozin (20 μM) and primaquine (300 μM). Samples were removed to the ice for various amounts of time and diluted in cold PBS to stop the surface antibody from further endocytosis. Samples were washed in PBS and analyzed by flow cytometry.

#### Recycling assay.

Cells were labelled as previously described with FITC-conjugated PD-L1 and washed in PBS. Cells were cultured in RPMI-1640 medium and incubated at 37°C for 30 minutes to allow antibody-labeled PD-L1 to undergo endocytosis. After washing, samples went through 2 rounds of resuspension in formulated low pH buffer (pH 2.5–2.8, 0.5 M NaCl, 0.5% acetic acid) for 2 minutes on ice to strip remaining surface bound antibody. Aliquots of cells were recultured in RPMI-1640 medium. Baseline samples were kept on ice and others were removed to 37°C in the presence or absence of canagliflozin (20 μM), as indicated in the figure legends. Samples were removed to the ice for various amounts of time and diluted in cold PBS to stop the surface antibody from further endocytosis. Samples were washed in PBS and analyzed by flow cytometry.

### IHC staining

For IHC staining, all tissue specimens were stained with 3% hydrogen peroxide (ZSGB-BIO) after deparaffinization and blocked by incubating with blocking buffer containing 5% goat serum (Gibco). The specimens were treated with antibodies against PD-L1 (1:100) or SGLT2 (1:350) at 4°C overnight. Specimens were then incubated with HRP-conjugated secondary antibodies (ZSGB-BIO), followed by treatment with an avidin-biotin-peroxidase complex and were developed with 3, 3-diaminobenzidine as per manufacturer’s protocol. All immunostained slides were scanned on Image-Pro Plus 6.0 software (IPP, version 6.0, Media Cybernetics) for quantification by digital image analysis. The expression of targeted protein in tumor tissue specimens was calculated from both the intensity of the immunostaining — as “–” for negative staining (0%), “+” for weak staining (1%–24%); “++” for intermediate staining (25%–49%); and “+++” for strong staining (>50%) (6), and the percentage, ranging from 0%–100% of immune-positive cells. A histo score of less than 50% was considered low expression and greater than 50% was considered high expression, as previously described (49); statistics were analyzed using the Pearson correlation test.

### Immunofluorescence

Cells were seeded in an 8-well chamber slider (Thermo Fisher Scientific) at approximately 50% confluence, with or without plasmid transfection. Cells were washed in ice-cold PBS and fixed with 4% formaldehyde for 20 minutes. Cells were permeabilized with 0.1% Triton X-100 in PBS at 4°C for 10 minutes, followed with blocking in 3% BSA in PBS at room temperature for 30 minutes. After blocking, the cells were incubated with primary antibodies at 4°C overnight. Secondary antibodies conjugated with Alexa Fluor 562 (1:200, A10042, Life Technology) or Alexa Fluor 488 (1:200, Life Technology, A21206). Followed by staining with DAPI (1:10000, Southernbiotech). The slides were imaged on an Olympus Fluoview-microscope (FV10i-O).

### Protein half-life assays

Cells were plated on 6-well plates and after 12 hours, cells were treated with cycloheximide (10 μg/mL) or canagliflozin (20 μM) + Cycloheximide (10 μg/mL) for different time points. Alternatively, cells were transfected with NC siRNA or SGLT2 siRNA under indicated conditions. After 24 hours transfection, cycloheximide (10 μg/mL) was added into these 2 groups for different time points. Cells were collected immediately and lysed, protein content was measured by immunoblot analysis and Image J was used for grayscale analysis.

### RNA isolation and quantitative real-time PCR

Cells were seeded in 6-well plates in the presence or absence of canagliflozin (20 μM) and IFN-γ (10 ng/mL), as indicated in the figure legends. Total RNA was extracted used TRIzol Reagent (TaKaRa) and quantified by the NanoDropND-1000 Spectrophotometer (Thermo Fisher Scientific). cDNA was synthesized from 2 μg purified total RNA with TransScript 1-Step gDNA Removal and cDNA Synthesis SuperMix (TransGen Biotech) according to the manufacturer’s instructions. Quantitative PCR was performed with iTaqTM Universal SYBR Green Supermix (Bio-Rad), in 7500 Fast Dx Real-Time PCR Instrument (Applied Biosystems). Quantification was calculated using the comparative Ct method and was presented as fold change. HuPD-L1 mRNA levels were normalized to Huβ-Actin mRNA levels. Primers pairs used for quantitative real-time PCR are as follows: human *CD274*: forward, 5′-TCACTACACAGCCCTCCTAA-3′, reverse, 5′-ACACCAGAATATGGCCAAGAG-3′; human *ACTB*: forward, 5′-ATTCCTATGTGGGCGACGAG-3′, reverse, 5′-CCAGATTTTCTCCATGTCGTCC-3′.

### siRNA-mediated silencing

Cells were seeded in 6-well plates and cultured for 24 hours, followed by transfection with the transfected reagent JetPRIME (Polyplus) and target siRNA (SGLT2, SGLT1, GLUT1, SPOP, STUB1, or β-TRCP) or scrambled siRNA (negative control [NC]), according to the manufacturer’s instructions. The siRNA sequences used are listed in Table 1.

### CRISPR/Cas9-mediated KO of PD-L1

Through CDS analysis of human and mouse *CD274* genes, exon 2 and 3 were determined to be the knockout position. Using CRISPR design tool (http://crispor.tefor.net/), 4 targeted sgRNAs were obtained. The PX458-*CD274* sgRNAs were generated by inserting the targeted sgRNAs into Bbs1-digested PX458 plasmid (Addgene), which were further confirmed by sequencing. To generate PD-L1 KO H1299 and CT26 cells, H1299 and CT26 cells were electroporated with PD-L1 KO plasmids or control plasmid as previously described (50). Briefly, H1299 and CT26 cells were seeded into a 5 cm dishes with RPMI-1640 medium containing 10% FBS. Cells were electroporated using indicated transfection regent after reaching 60%–70% confluence. Clones derived from single PD-L1 KO cells were obtained by fluorescence-activated cell sorting using FACSAria II (BD Biosciences) in a 96-well plate, and the successfully edited clones were determined using Western blotting. The gRNA oligonucleotide sequences are as follows: Mouse *Cd274* gRNA1: forward: 5′-CACCGTCACCACTTCCCGGACAGAG-3′, reverse: 5′-AAACCTCTGTCCGGGAAGTGGTGAC-3′; Mouse *Cd274* gRNA2:forward: 5′-CACCGAACTAATATGTCAGGCCGA-3′, reverse: 5′-AAACTCGGCCTGACATATTAGTTC-3′; Human *CD274* gRNA1: forward: 5′-CACCGGTTCCCAAGGACCTATATG-3′, reverse: 5′-AAACCATATAGGTCCTTGGGAACC-3′; Human *CD274* gRNA2: forward: 5′-CACCGACTGCTTGTCCAGATGACTT-3′, reverse: 5′-AAACAAGTCATCTGGACAAGCAGTC-3′.

### Plasmid construction

Cells were transfected with the indicated plasmids or empty vector by using transfected reagent JetPRIME (Polyplus) according to the manufacturer’s instructions. The expression vector PD-L1-HA was generated by inserting PCR-amplified *PD-L1* cDNA which was synthesized by using KOD-Plus-Neo kit (Toyobo) according to the manufacturer’s instructions into the pcDNA3.0-HA vector. Similarly, PD-L1-Flag, SGLT2-Flag, and SPOP-Flag were generated by inserting the indicated cDNA into pcDNA3.0-Flag vector. The SGLT2-R300A and SGLT2-S392A/S393A mutations were performed by site-directed mutagenesis. To generate PD-L1-RFP, SGLT2-GFP, and the PD-L1 and SGLT2 deletion mutant, PCR splicing was used. The primers we used for site-directed mutagenesis and PCR splicing were listed in Table 2.

### Lentiviral production and transduction

HEK293FT cells were used for packaging of lentivirus, and subsequent infection of various cell lines were performed. Cells with 90% confluence were transfected using linear polyethylenimine hydrochloride (Sigma-Aldrich) in Opti-MEM medium (Invitrogen), and packaged plasmids P8.9, VSVG, and shRNA at a 5:1:5 ratio. The viral supernatant was collected 48 hours after transfection and filtered through a 0.45 μm filter. Viruses were used to infect cells, combined with 4 μg/mL polybrene (Sigma-Aldrich). For lentiviral expression of shRNA: shSGLT2, shSGLT1, or nontargeting control shRNA were cloned into the pLKO.1 vector. shRNA targeted sequences are listed here: human *SLC5A1* forward, CCGGACAGCAAAGAGGAGCGTATTGCTCGAGCAATACGCTCCTCTTTGCTGTTTTTTTG; human *SLC5A2#1* forward, CCGGCCTAGTCATTGCTGCATATTTCTCGAGAAATATGCAGCAATGACTAGGTTTTTG; human *SLC5A2#2* forward, CCGGCCTAGTCATTGCTGCATATTTCTCGAGAAATATGCAGCAATGACTAGGTTTTTG.

### Cell proliferation assay

H292 cells were infected with shSGLT2 or nontargeting control shRNA before they were seeded in 96-well plates at a density of 3,000 cells per well. The sulforhodamine B (SRB) assay was used. Cells were fixed with 10% (w/v) trichloroacetic acid at indicated times and stained for 30 minutes at room temperature. The excess dye was washed with 1% (v/v) acetic acid and followed by dissolving protein-bound dye in 10 mM Tris-base. OD values were measured at 540 nm by microplate reader (Molecular Devices).

### Animal experiments

To study the effect of canagliflozin as well as SGLT2 on tumorigenesis, we performed 2 different animal models (51, 52).

### In vivo experiment in the CT26 mouse tumor model

CT26 cells (4 × 10^5^ per mouse) were injected subcutaneously in 100 μL medium into 6-week-old BALB/c female mice. Tumor volumes were measured every 3 days with a caliper and calculated by the formula: length × width^2^ × 0.5. Mice were pooled and randomly divided into 5 groups with comparable average tumor size, and were grouped into control, canagliflozin (Selleck), anti-mouse PD-L1 treatment (BE0101, BioXcell), anti-mouse CTLA-4 treatment (BE0131, BioXcell) and canagliflozin plus anti-CTLA-4 antibody treatment. Control and canagliflozin were treated daily with canagliflozin (50 mg/kg) or rat IgG2a isotype control (BE0089, BioXcell) only; anti-PD-L1 and anti-CTLA-4 antibody treatment were given by intravenous injection (5 mg/kg) twice a week. Mice were euthanized 21 days after drug treatment or if the tumor volume exceeded 2,000 mm^3^. Blood samples were immediately harvested retroorbitally.

### In vivo humanized immune-transformation model experiment

4-week-old NSG (NOD-*Prkdc^scid^Il2rg^em1^/Smoc*) male mice were purchased from Shanghai Model Organism Center Inc. for generation of humanized mice. Mice were bred and maintained under specific pathogen-free conditions at the Center for Drug safety Evaluation and Research of Zhejiang University. NSG mice were tail vein injected with activated human PBMC (1.5 × 10^7^ per mouse), which was purchased from AllCells Biotech Shanghai Co. Ltd.; successful humanization of each mouse was quantified from mouse peripheral blood via flow cytometry using anti-hu-CD45^+^. CD34^+^ humanized mice, which were constructed by transportation of human umbilical cord blood and fetal liver hematopoietic stem cells, were purchased from All Cells Biotech Shanghai Co. Ltd. H292 cells (5 × 10^6^) and shSGLT2 H292 cells (5 × 10^6^) were injected subcutaneously in 100 μL medium into the right flank of NSG mice. Tumor volume was measured with a caliper and when the tumor reached roughly 100 mm^3^, mice were pooled and randomly divided into 3 groups with comparable average tumor size. Mice were grouped into control, canagliflozin, and anti-PD-1 antibody treatment (Nivolumab, Opdivo). The canagliflozin group was treated daily with canagliflozin (50 mg/kg), and anti-PD-1 antibody treatment were given by intravenous injection (5 mg/kg) twice a week for a total of 3 injections. Mice were euthanized a week after drug treatment and blood samples were harvested retroorbitally.

Metabolite analysis

H292 cells for metabolite analysis were seeded in 10 cm dishes with RPMI-1640 medium containing 10% FBS. Cells were treated with canagliflozin (20 μM) or DMSO alone (control) for 24 hours. For another group, cells were transfected with GLUT1 or NC siRNA for 24 hours. Cells were washed with cold PBS 3 times gently and resuspended in 1 ml 80% methanol at –80°C for 1 hour. The samples were thawed at room temperature and centrifuged for 20 minutes (14,000*g*, 4°C). The supernatant was dried in a vacuum centrifuge. For liquid chromatography-mass spectrometry (LC-MS/MS) analysis, the samples were processed by Applied Protein Technology. Briefly, the samples were redissolved in 100 μL acetonitrile/water 1:1, (v/v) and adequately vortexed. Centrifuged for 15 minutes (14,000*g*, 4°C). The metabolites of glycolysis pathway were measured by LC-MS/MS and the values of key components, such as fructose 1,6-bisphosphate, glucose-6-phosphate, and lactate shown in the results were percentages relative to NC.

### Statistics

Data in the bar graphs are presented as the mean ± SD and represent fold change or percentage in relation to control or untreated groups with 3 independent experiments. GraphPad Prism v.6.0 was used for statistical analysis. Unpaired 2-tailed Students’ *t* tests were performed to determine statistical significance of experimental data, with *P* < 0.05 being considered statistically significant. Representative immunofluorescence results were based on SD of 3 independent cell culture experiments, and at least 3 images were collected in each replicate. For multiple group tumor growth analysis, 1-way ANOVA was used to test an overall difference at each group data collection time point. For the survival data analysis of patients with NSCLC, preclinical patients with different PD-L1 and SGLT2 expression levels were tested by the Kaplan-Meier method and Gehan-Breslow-Wilcoxon to detect differences in survival curves between groups.

### Study approval

All tissue samples were collected in compliance with informed consent policy. The study protocol was approved by the Institutional Review Board at Zhejiang Cancer Hospital Committee in Hangzhou (China) and all patients provided written informed consent (IRB-2019-175 and IRB-2022-540). For animal studies, we have complied with the guidelines approved by the Institutional Animal Care and Use Committee of Zhejiang University in Hangzhou (IACUC no.19-136, 19-262). All animal procedures were performed according to the guidelines approved by the Institutional Animal Care and Use Committee of Zhejiang University. All experiments were conducted with protocols approved by the Center for Drug Safety Evaluation and Research of Zhejiang University.

## Author contributions

LD and XC designed and wrote the study. XC designed, performed and analyzed the in vivo and in vitro experiments. XD, WZ, and HG assisted with the Western blotting and in vivo experiments. MY, XG, XX, JW, and SL assisted with cell culture; YX and JF contributed to the collection of patient samples. XP and HW analyzed data; JC assisted in editing the manuscript, and QH and BY conceptualized the work. LD and XC contributed to this manuscript equally, so the first author was determined alphabetically.

## Supplementary Material

Supplemental data

## Figures and Tables

**Table 2:**
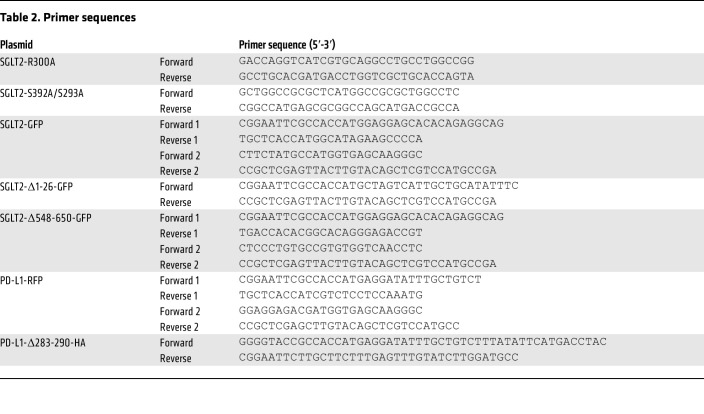
Primer sequences

**Table 1:**
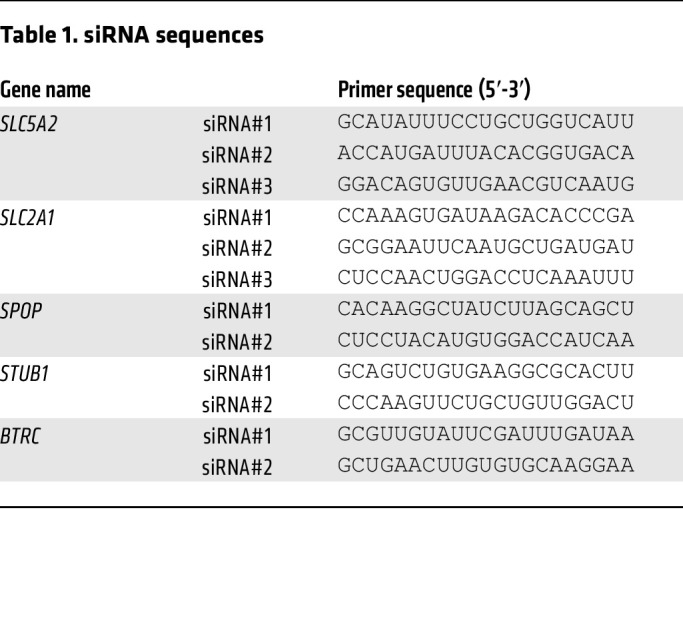
siRNA sequences

## Version 1 01/03/2023

Electronic publication
