# Supplementary material for: Canagliflozin primes antitumor immunity by triggering PD-L1 degradation in endocytic recycling
Source: J Clin Invest. 2023 Jan 3;133(1):e154754. doi: 10.1172/JCI154754 (PMC9797339; doi:10.1172/JCI154754)
Supplement: Supplemental data [file jci-133-154754-s104.pdf]

Extend Data

A

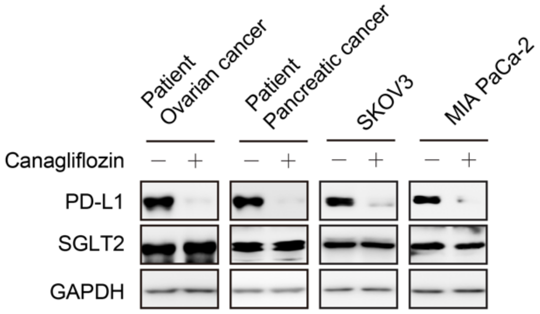

Extend Figure 1 The impact of Canagliflozin on PD-L1 expression in tumor cells. A, Canagliflozin decreased the expression of PD-L1 on ovarian cancer cell line SKOV3, pancreatic cancer cell line MIA PaCa-2, primary ovarian cancer and pancreatic cancer patient-derived cancer cells, blots were run in parallel.

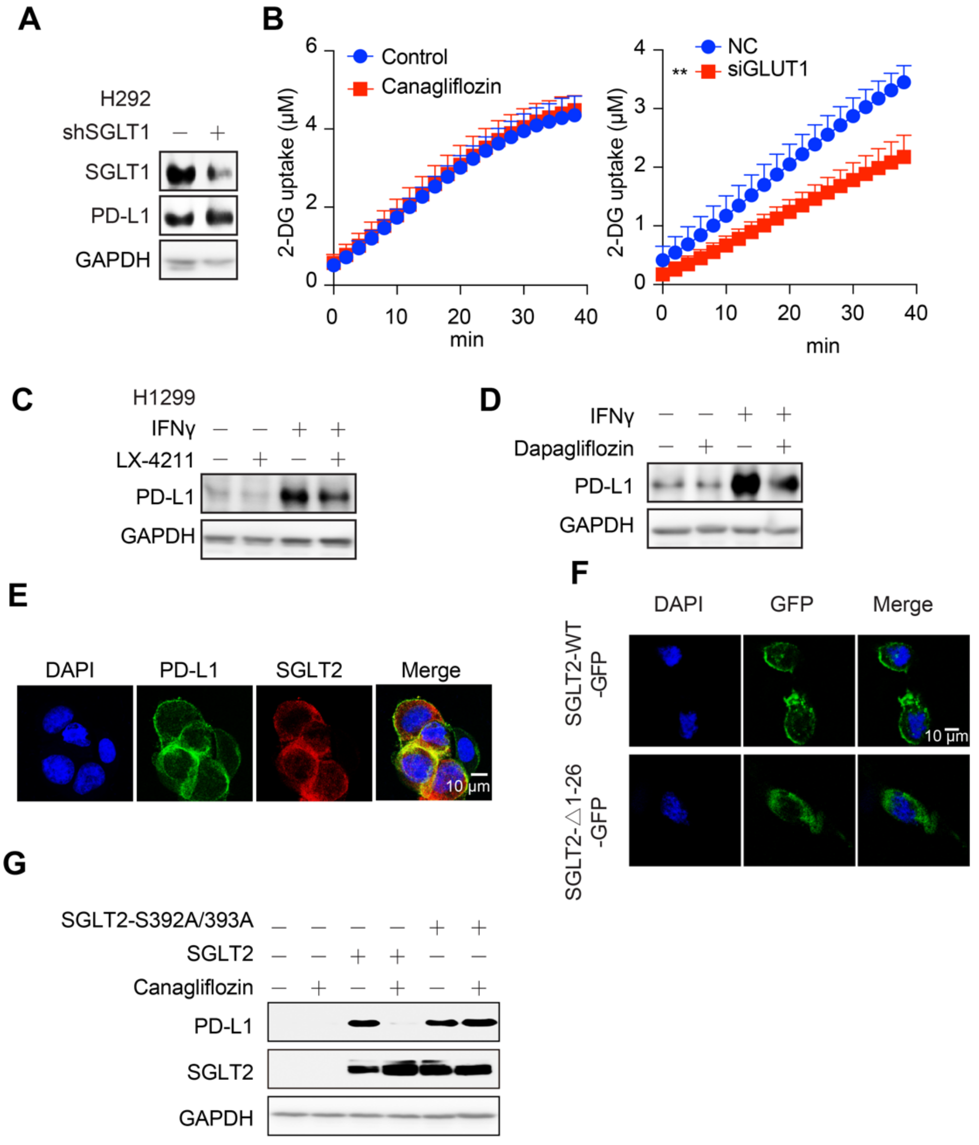

Extend Figure 2 Canagliflozin caused PD-L1 decrease is an on-target effect. A, SGLT1 was knocked down by shRNA and PD-L1 expression was analyzed, blots were run in parallel. **B,** 2-DG uptake was not significantly affected by Canagliflozin at the concentration (20 μM) we use, the reduction in 2-DG uptake was observed in siGLUT1 groups. **C, D,**NSCLC cell line H1299 was treated with various SGLT2 inhibitors LX-4211, Dapagliflozin (20 μM) alone or together with IFNγ (10 ng/mL) for 24 hours and PD-L1 expression was analyzed by Western blotting. **E,** Localization of endogenous SGLT2 and endogenous PD-L1 was determined by confocal analysis. Scale bar: 10 μm. **F,** N-terminal

26-amino-acid sequence was required for plasma membrane localization of SGLT2. Cells were transfected with SGLT2-WT-GFP or SGLT2-△1-26-GFP for 24 hours, and the localization of SGLT2 was determined by Immunofluorescence. Scale bar: 10 μm. **G,** Downregulation of PD-L1 caused by Canagliflozin was abolished when SGLT2 sodium-binding site was mutated. In H460 cell line, the expression of SGLT2 was first knocked down, then re-overexpressed the SGLT2-WT and SGLT2-S392A/393A using plasmid transfection, and then H460 cells were treated with Canagliflozin (20 μM) for 24 hours after transfected with SGLT2 or SGLT2-S392A/393A plasmids. The expression of PD-L1 was examined by Western blotting, blots were run in parallel. Statistical significance was determined by unpaired Students' *t* test (2-tailed). **, $p < 0.01$.

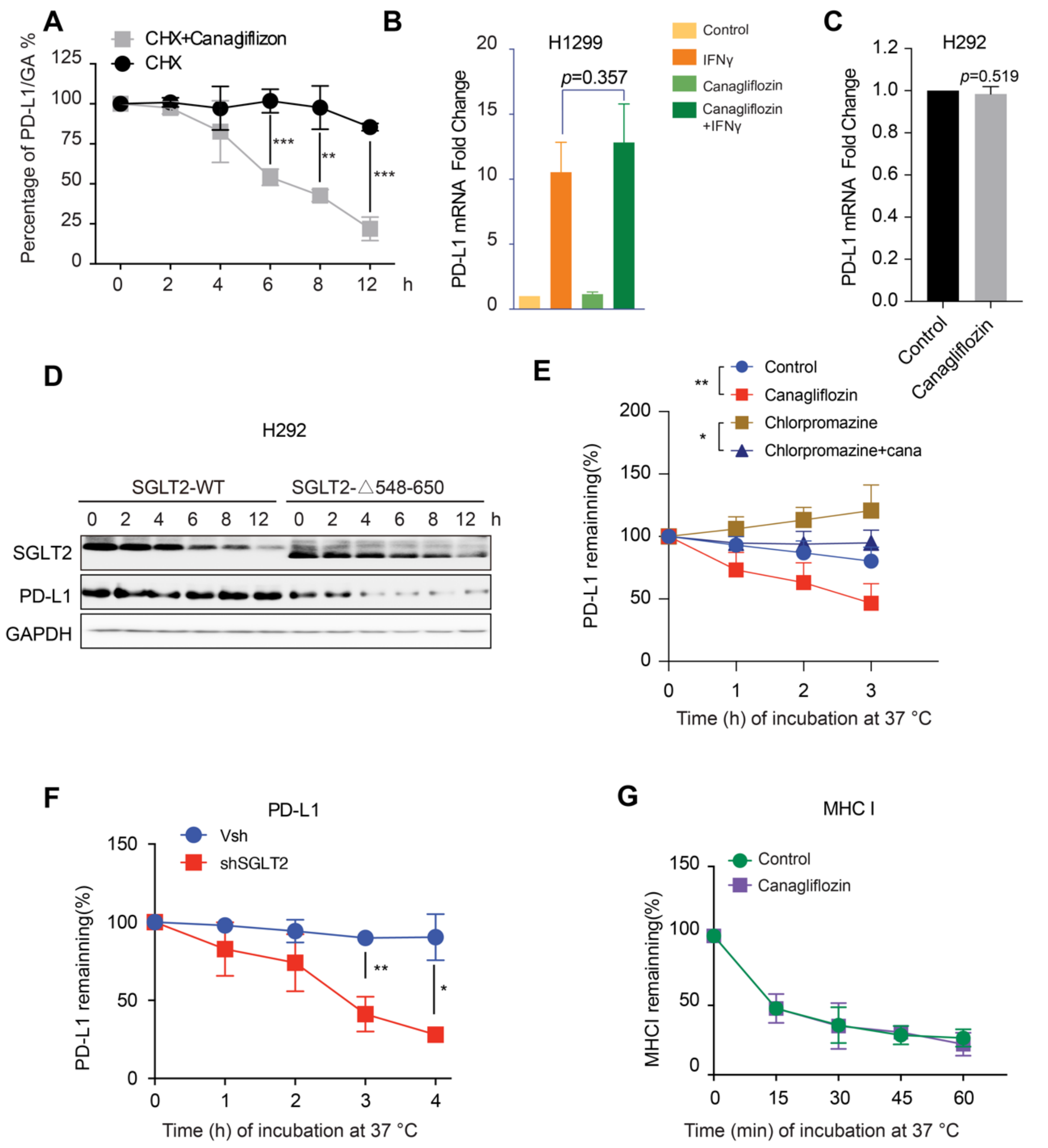

Extend Figure 3 Canagliflozin induced PD-L1 degradation. A, H1299 cells were treated with IFNγ (10 ng/mL) for 24 hours first, and then treated with Canagliflozin (20 μM) and Cycloheximide (10 μg/mL) for indicated time. The data are presented as the mean ± SD of triplicate experiments. **B,C,** *CD274* mRNA expression was not affected by Canagliflozin. H1299 cells were treated with IFNγand Canagliflozin (H292 were treated with Canagliflozin) as indicated for 24 hours and the *CD274* mRNA expression was determined by qRT-PCR. **D,** In H292 cell line, the expression of SGLT2 was first knocked down, then re-

overexpressed the SGLT2-WT and SGLT2-△548-650 using plasmid transfection, finally the protein half-life of PD-L1 was measured. SGLT2 absence caused decrease in half-life of PD-L1 was reversed by SGLT2-WT over-expression, While over-expression of SGLT2-△548-650 failed to prolonging the stability of PD-L1. **E,** Surface PD-L1 proteins were labelled with PD-L1 antibody at 4 °C for 2 hours, and then tumor cells were incubated at 37 °C in the presence or absence of Canagliflozin (20 μM) for 0, 1, 2, 3 hours. As for group endocytosis inhibitor and endocytosis inhibitor + Canagliflozin, tumor cells were incubated at 37 °C in the presence of endocytosis inhibitor (Chlorpromazine) of endocytosis for 1 hour. After finishing the above preprocessing, tumor cells were incubated at 37 °C in the presence or absence of Canagliflozin (20 μM) and Chlorpromazine for indicated time. Four parallel experiments were conducted. Canagliflozin did not cause PD-L1 degradation without PD-L1 internalized in the presence of endocytosis inhibitor. **F,** SGLT2 silencing induced cell surface PD-L1 degradation. **G,** Canagliflozin did not influence the surface expression of MHC I. Statistical significance was determined by unpaired Students' *t* test (2-tailed). *, $p < 0.05$; **, $p < 0.01$; ***, $p < 0.001$.

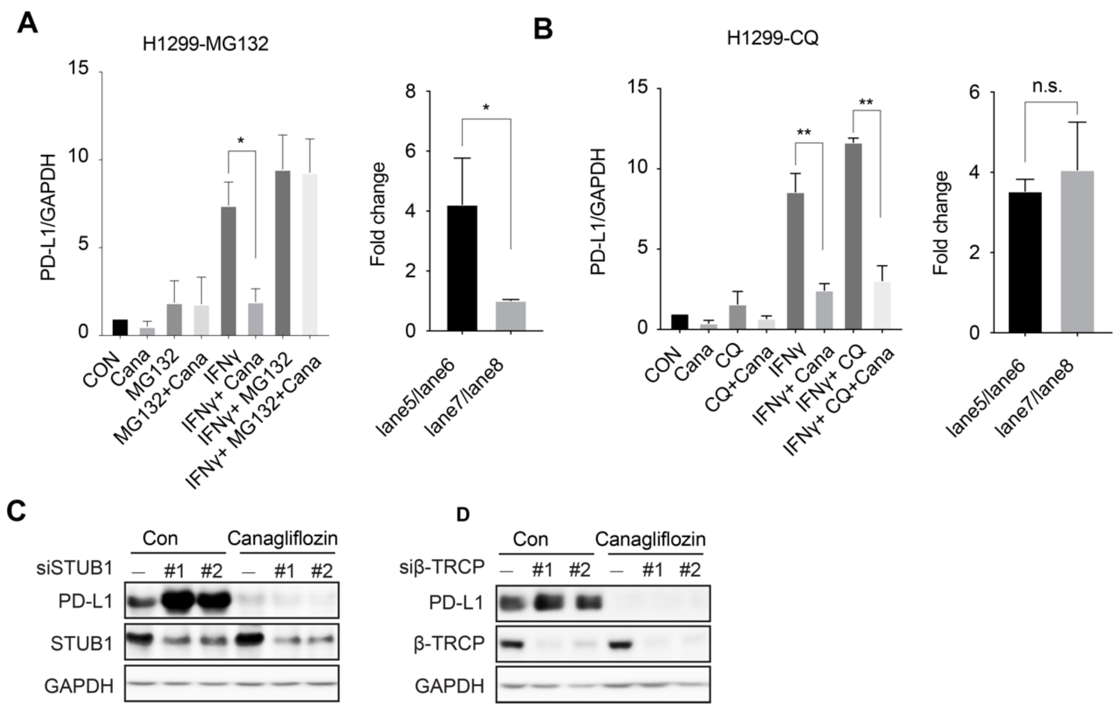

Extend Figure 4 Canagliflozin induced PD-L1 degradation through proteasome-dependent pathway. A, B, The PD-L1 expression after MG132 or CQ treatment was analyzed by Western blotting and was quantified using Image J grayscale analysis. The data are presented as the mean ± SD of triplicate experiments. **C,** Canagliflozin decreased PD-L1 expression when STUB1 E3 ligase was deleted by siRNA, blots were run in parallel. **D,** Canagliflozin decreased PD-L1 expression in the absence of β-TRCP E3 ligase, blots were run in parallel.. Statistical significance was determined by unpaired Students' *t* test (2-tailed). *, $p < 0.05$; **, $p < 0.01$; n.s. not significant.

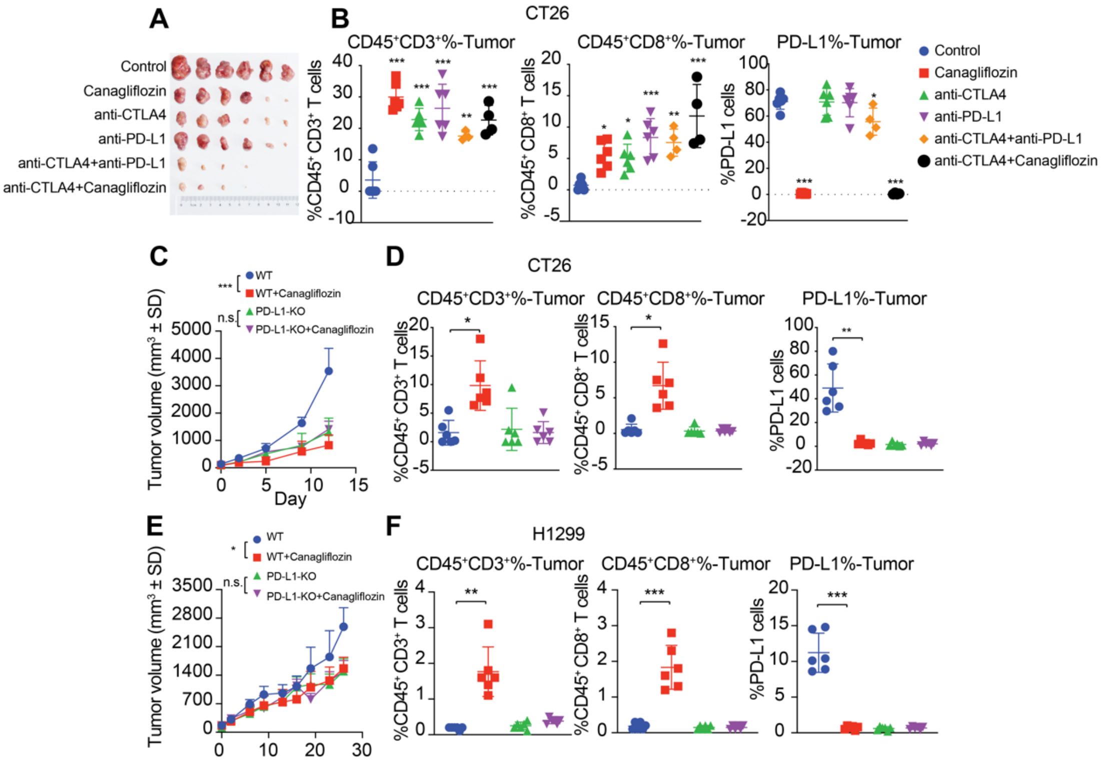

Extend Figure 5 Canagliflozin suppressed tumor mainly through suppression of PD-L1 and the existence of immune system. A, The combination of anti-PD-L1 and anti-CTLA4 antibodies achieved a comparable therapeutic efficacy compared anti-CTLA4 + Canagliflozin group. Tumor volume of CT26 cells in immunocompetent BALB/c mice treated with Canagliflozin, anti-PD-L1 mAb, anti-CTLA4 mAb, combination of Canagliflozin and anti-CTLA4 mAb, or combination of anti-PD-L1 mAb and anti-CTLA4 mAb. Data represent mean ± SD. *n* = 6 mice per group. **B,** Tumor-infiltrating $CD45^+CD3^+$ T cells and $CD45^+CD8^+$ T cells were detected by FACS. PD-L1 level in extracted tumor tissues was evaluated by FACS, data represent mean ± SD. Statistical significance was determined by one-way ANOVA with Dunnett's post hoc test. **C, D, E, F,** The anti-tumor effect of Canagliflozin was abolished when PD-L1 of CT26 and H1299 cell lines were knocked out by CRISPR/Cas9-mediated genome editing. Tumor-infiltrating $CD45^+CD3^+$ T cells

and $CD45^{+}CD8^{+}$ T cells were detected by FACS. PD-L1 level in extracted tumor tissues was evaluated by FACS, data represent mean ± SD. $n = 6$ mice per group. Statistical significance was determined by one-way ANOVA with Dunnett's post hoc test. *, $p < 0.05$; **, $p < 0.01$; ***, $p < 0.001$.

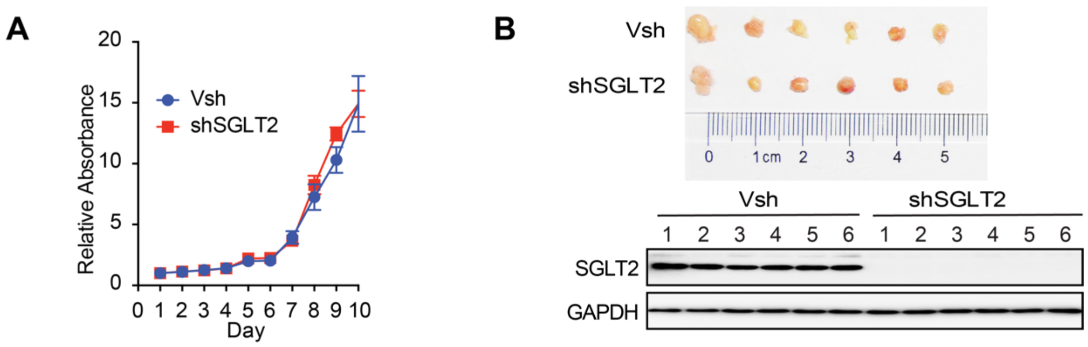

Extend Figure 6 SGLT2 knock down suppressed tumor growth in PBMC humanized H292 xenograft model. A, The depletion of SGLT2 had no effect on H292 cell proliferation. H292 cells were seeded in 96-well plates after infected with shSGLT2 or non-targeting control of shRNA, and then fixed with 10% (w/v) trichloroacetic acid at indicated times, and the cell proliferation was analyzed by SRB assay. Data represent mean ± SD. **B,** In NSG mice model without PBMC infusion, tumor growth was not significantly affected by SGLT2 knocked down. Tumor growth of H292 cells with (or without) SGLT2 knock down in NSG mice without PBMC injection. Data represent mean ± SD. *n* = 6 mice per group. Protein lysates from tumors were analyzed by Western blotting with SGLT2 and GAPDH antibodies.

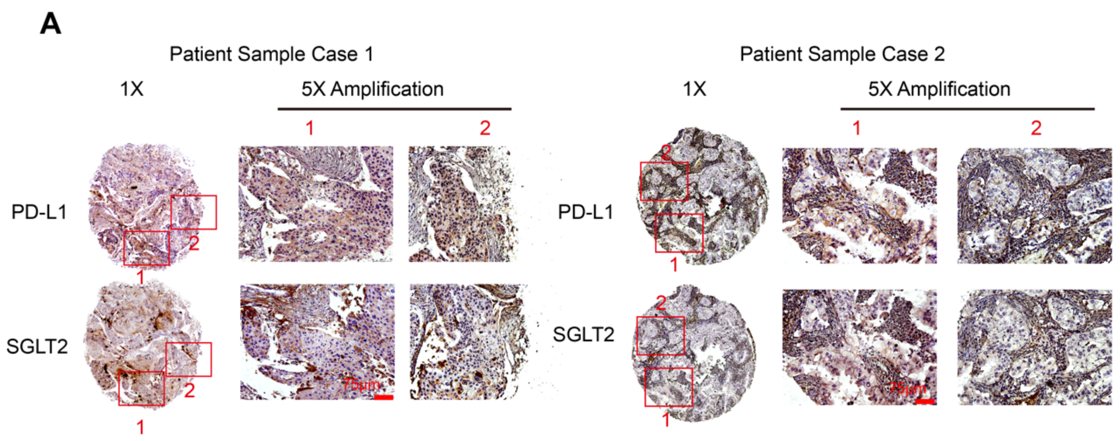

Extend Figure 7 SGLT2 positively correlated with PD-L1 expression in specimens of Lung cancer. A, Representative images of IHC staining of PD-L1 and SGLT2 in human Lung cancer tissues (*n*=2) are shown. The picture on the right is a partial magnification of the picture on the left by 5 times. Scale bar:75μm.

Table S1 Information of small molecule drug library, related to Figure 1.

Number	Drug	Number	Drug	Number	Drug
1	Vigabatrin Hydrochloride	34	Tolnaftate	67	Tosylate Monohydrate
2	LCZ696	35	Pyridinealdoxime	68	Montelukast sodium
3	Tyloxapol	36	Rasagiline	69	WY-14643 (Pirinixic Acid)
4	Pargyline	37	Betamethasone 17-valerate	70	Trelagllptin succinate
5	Risedronic Acid	38	Papaverine hydrochloride	71	Indacaterol Cilastatin
6	Etamsylate	39	Clopidogrel sulfate	72	Cilastatin
7	Adiphenine hydrochloride	40	Racecadotril	73	Etonogestrel
8	Procainamide hydrochloride	41	Milnacipran hydrochloride	74	Metformin
9	Molsidomine	42	Erdosteine	75	Aclidinium bromide
10	Brinzolamide	43	Tanshinone Ⅰ	76	Nafanpstat mesylate
11	Roxatidine Acetate hydrochloride	44	Isoliquirigenin	77	Fimasartan (B-R-A-657)
12	Pranoprofen	45	Allopurinol	78	Danazol
13	Nylidrin hydrochloride	46	Aptal	79	Clinofibrate
14	Ticagreler	47	Benfluorex hydrochloride	80	Nicorandil
15	Prednisolone phosphate sodium	48	Sulfadoxine	81	Azatadine Maleate
16	Amlodipine Besylate	49	Methimazole	82	Aprepitant

17	Amcinonide	50	L-Thyroxine	83	Canagliflozin
18	Dimenhydrinate	51	Amprolium	84	Obeticholic Acid
19	Methylprodnisolone Sodium Succinate	52	Torasemide	85	Ataluren (PTC124)
20	Canrenone	53	Memantine hydrochloride	86	JQ1
21	Aminoguanidine hydrochloride	54	Captoprii	87	Macitentan
22	Leucobasal	55	indapamide	88	PeraMpanel
23	Diammonium Glycyrrhizinate	56	Rimpnabant hydrochloride	89	Ivacraftor
24	Metyprapone	57	Sodium Phenylbutyrate	90	MK-4305
25	Aminobenzen esulfonamide	58	Montelukast	91	Trans-Tranilsat
26	Pioglitzone hydrochloride	59	Varenicline Tartrate	92	Ramatroban
27	Pyridoxine	60	Cinacalcet hydrochloride	93	Conivaptan hydrochloride
28	Medrysone	61	Desvenlafaxine	94	Maraviroc
29	Ergotamine bitartrate	62	Acetohydroxamic acid	95	Selexipag (NS-304)
30	Choline chloride	63	Ezetimibe	96	Valsartan
31	Risedronate Sodium	64	Fasudil	97	Piribedil
32	Capsaicin	65	Rivastigmine tartrate	98	Delapril hydrochloride
33	Ketanserin	66	Amitraz	99	Istradefylline

Table S2 Clinicopathologic characteristics of Lung patient tissues microarray cohorts, related to Figure 8.

Patient No.	Gender	Age	Histotype	TNM	PFS (months)	OS (months)	Metastatic Sites
1	male	54	SCLC	T2N0M0	0	75	brain
2	male	68	LUSC	T2N2M0	15	17	brain
3	male	47	LUAD	T2N1M0	9	35	brain, lung
4	male	70	LUSC	T3N0M0	10	29	brain
5	female	57	LUAD	T2N0M0	10	24	brain
6	male	49	LUSC	T2N1M0	5	108	brain
7	male	55	others	T2N0M0	16	19	pleural
8	male	65	LUSC	T4N0M0	3	9	lung
9	male	62	SCLC	T2N2M0	70	70	0
10	female	49	LUAD	T4N2M1	25	29	hydrothorax
11	male	58	LUAD	T1N2M0	7	22	brain
12	male	65	others	T2N0M0	15	20	lung
13	male	51	LUSC	T2N0M0	13	61	throat
14	female	61	LUAD	T2N1M0	31	34	bone
15	male	70	LUSC	T2N1M0	25	25	0
16	male	63	LUSC	T2N0M0	50	50	0
17	female	64	LUAD	T1N0M0	91	91	0
18	male	66	LUSC	T2N0M0	90	90	0
19	female	54	LUAD	T2N0M0	13	90	brain
20	female	57	LUAD	T2N0M0	77	77	0
21	male	58	LUAD	T2N0M0	90	90	0
22	male	72	LUAD	T2N0M0	15	28	chest wall
23	male	61	LUSC	T2N0M0	40	90	mediastinum
24	male	63	LUSC	T2N0M0	90	90	0
25	male	64	LUSC	T2N0M0	1	53	lung
26	male	73	LUSC	T2N0M0	89	89	0
27	male	55	LUSC	T3N0M0	17	17	0
28	male	75	LUSC	T3N0M0	2	4	throat, bone
29	male	52	LUSC	T3N2M0	12	12	0
30	male	73	LUAD	T2N0M0	59	59	lung
31	female	54	LUAD	T2N0M0	89	89	0
32	male	53	LUSC	T4N1M0	5	5	0
33	male	63	LUSC	T2N0M0	76	76	0
34	female	63	LUAD	T2N0M0	17	51	brain
35	male	56	LUSC	T4N2M0	4	4	0
36	male	67	LUAD	T2N2M0	3	27	bone
37	male	75	LUSC	T1N0M0	19	71	lung

38	male	67	LUAD	T2N0M0	51	51	0
39	male	70	others	T3N2M0	2	12	lung
40	male	39	LUAD	T4N2M0	9	29	brain, lung
41	male	71	LUAD	T2N0M0	12	27	lung, bone
42	male	54	LUSC	T4N1M0	3	3	0
43	male	58	LUSC	T2N1M0	4	48	mediastinum
44	female	76	LUAD	T2N1M0	9	10	lung
45	female	59	LUAD	T1N2M0	15	18	mediastinum
46	male	55	LUAD	T1N0M0	74	74	0
47	male	77	LUAD	T1N0M0	18	20	brain, lung
48	female	63	LUAD	T1N2M0	51	51	brain
49	male	56	LUSC	T4N1M0	87	87	0
50	male	54	LUAD	T1N0M0	87	87	0
51	male	58	LUAD	T4N2M0	37	87	bone, brain
52	male	70	LUAD	T1N0M0	15	74	lung
53	female	52	SCLC	T2N0M0	20	36	brain
54	female	51	LUSC	T2N1M0	86	86	0
55	female	62	LUAD	T2N2M0	21	14	brain
56	male	65	LUAD	T2N0M0	37	76	lung
57	male	60	LUAD	T4N1M0	9	42	abdomen
58	male	67	LUSC	T3N1M0	4	14	lung
59	male	58	LUSC	T2N1M0	18	18	0
60	male	70	LUSC	T2N0M0	37	42	liver
61	male	67	LUSC	T2N1M0	85	85	0
62	male	50	LUAD	T1N0M0	84	84	0
63	male	66	LUSC	T3N1M0	2	71	rendal
64	female	60	LUAD	T2N0M0	9	72	lung, bone
65	female	45	LUAD	T2N2M0	84	84	0
66	male	53	LUSC	T2N2M0	11	15	lung
67	male	56	LUSC	T2N0M0	71	71	0
68	female	41	LUAD	T2N0M0	64	64	0
69	male	61	LUAD	T2N1M0	7	18	lung
70	female	65	LUAD	T1N2M0	76	76	0
71	male	56	LUAD	T2N1M0	76	76	0
72	male	52	LUSC	T2N1M0	35	35	0
73	male	68	SCLC	T1N0M0	40	40	0
74	female	73	LUSC	T1N0M0	76	76	0
75	female	45	LUAD	T2N1M0	45	51	brain
76	male	61	LUAD	T2N2M0	9	9	bone, mediastinum
77	male	59	LUSC	T3N0M0	5	7	abdomen
78	female	72	LUAD	T2N0M0	17	17	0

79	male	64	LUSC	T2N1M0	75	75	0
80	male	71	LUAD	T2N0M0	74	74	0
81	male	51	LUSC	T2N0M0	8	74	liver
82	female	59	LUAD	T2N0M0	61	61	0
83	female	56	LUSC	T2N2M0	7	7	lung, liver
84	female	63	others	T1N2M0	61	61	0
85	male	65	LUAD	T2N0M0	5	6	lung
86	female	63	LUAD	T2N2M0	5	54	brain
87	male	53	LUAD	T3N0M0	37	37	0
88	male	56	LUSC	T2N0M0	74	74	0
89	male	53	LUSC	T2N1M0	74	74	0
90	male	66	LUAD	T2N2M0	61	61	0
91	male	61	LUSC	T1N0M0	37	37	0
92	male	64	LUAD	T2N0M0	9	37	lung
93	male	53	LUAD	T4N0M0	19	29	brain
94	female	61	LUAD	T2N0M0	37	37	0
95	male	68	LUAD	T2N1M0	1	1	0
96	male	67	LUAD	T3N0M0	24	24	0
97	male	66	LUAD	T2N0M0	60	60	0
98	female	72	LUSC	T2N0M0	6	6	0
99	male	60	LUAD	T2N0M0	24	24	0
100	female	62	LUSC	T2N0M0	60	60	0

PFS, progression-free survival; OS, overall survival. LUSC: Lung squamous cell carcinoma. LUAD: Lung adenocarcinoma. TNM stage based on *The 8th Edition Lung CancerStage* Classification.

Table S3 Clinicopathologic characteristics of PD-1 mAb monotherapy cohorts, related to Figure 8.

Patient No.	Gender	Age	Response	PFS（months）	OS（months）	Tumor types
A9	Male	56	SD	1.8	4.7	LUAD
A16	Male	55	SD	1.6	5.5	LUSC
A6	Male	63	PR	4.1	5.9	LUSC
A8	Female	54	PR	5.5	12.0	LUSC
A15	Male	65	SD	4.3	12.3	LUAD
A12	Male	66	PD	1.3	12.4	LUSC
A14	Male	62	SD	5.6	13.0	LUSC
A3	Male	47	SD	15.4	20.5	LUAD
A7	Male	52	SD	14.8	20.5	LUSC
A10	Male	69	SD	14.2	21.0	LUSC
A5	Male	62	SD	12.3	21.1	LUSC
A13	Male	49	SD	21.3	21.2	LUSC
A2	Male	71	SD	24.8	24.6	LUAD
A11	Female	44	SD	10.5	24.8	LUAD
A1	Male	60	SD	23.4	25.3	LUSC
A4	Male	56	PR	25.1	27.0	LUSC

PD-1, programmed death-1; PR, partial response; SD, stable disease; PD, progressive disease; PFS, progression-free survival; OS, overall survival. Patients with CR, PR and SD > 6 months were classified as responders, while patients with SD ≤ 6 months and PD were classified as non-responders. LUSC: Lung squamous cell carcinoma. LUAD: Lung adenocarcinoma. TNM stage based on *The 8th Edition Lung CancerStage* Classification.

Table S4 Analysis of SGLT2 expression levels in NSCLC patients, related to Figure 8.

Patients tissue array	SGLT2		
	High	Low	Total
	6	10	16

The low expression category includes those whose positive staining rate is smaller than 50%, whereas the high expression category greater than 50%.

Table S5 Clinicopathologic characteristics of PD-1 mAb monotherapy cohorts, related to Figure 8.

	PD-1 mAb Monotherapy	
Patient Characteristics	Responders n = 11	Non-responders n = 5
Age (years), mean±SEM	57±2.659	60.8±2.267
Male	9	5
Female	2	0
Response, n (%)		
PR	3(27.3)	0
SD	8(72.7)	4(80.0)
PD	0	1(20.0)
Median PFS (months)	15.58±2.22	2.92±0.8576
12 months PFS (%)	72.7	0
Median OS (months)	20.35±1.875	9.58±1.837
12 months OS (%)	90.9	60

PD-1, programmed death-1; PR, partial response; SD, stable disease; PD, progressive disease; PFS, progression-free survival; OS, overall survival. Patients with CR, PR and SD > 6 months were classified as responders, while patients with SD ≤ 6 months and PD were classified as non-responders.
